# cDNA microarray analysis of bovine embryo gene expression profiles during the pre-implantation period

**DOI:** 10.1186/1477-7827-2-77

**Published:** 2004-11-24

**Authors:** Koichi Ushizawa, Chandana B Herath, Kanako Kaneyama, Satoshi Shiojima, Akira Hirasawa, Toru Takahashi, Kei Imai, Kazuhiko Ochiai, Tomoyuki Tokunaga, Yukio Tsunoda, Gozoh Tsujimoto, Kazuyoshi Hashizume

**Affiliations:** 1 Reproductive Biology and Technology Laboratory, Developmental Biology Department, National Institute of Agrobiological Sciences, 2 Ikenodai, Tsukuba, Ibaraki 305-8602, Japan; 2 Department of Genomic Drug Discovery Science, Graduate School of Pharmaceutical Sciences, Kyoto University, 46-29 Yoshida Shimoadachi-cho, Sakyo-ku, Kyoto 606-8501, Japan; 3 Development and Differentiation Laboratory, Developmental Biology Department, National Institute of Agrobiological Sciences, 2 Ikenodai, Tsukuba, Ibaraki 305-8602, Japan; 4 Laboratory of Animal Reproduction, College of Agriculture, Kinki University, 3327-204 Nakamachi, Nara 631-8505, Japan; 5 Department of Veterinary Medicine, Faculty of Agriculture, Iwate University, 3-18-8 Ueda, Morioka, Iwate 020-8550, Japan; 6 Department of Technology, National Livestock Breeding Center, 1 Odakurahara, Odakura, Nishigo, Fukushima 961-8511, Japan

## Abstract

**Background:**

After fertilization, embryo development involves differentiation, as well as development of the fetal body and extra-embryonic tissues until the moment of implantation. During this period various cellular and molecular changes take place with a genetic origin, e.g. the elongation of embryonic tissues, cell-cell contact between the mother and the embryo and placentation. To identify genetic profiles and search for new candidate molecules involved during this period, embryonic gene expression was analyzed with a custom designed utero-placental complementary DNA (cDNA) microarray.

**Methods:**

Bovine embryos on days 7, 14 and 21, extra-embryonic membranes on day 28 and fetuses on days 28 were collected to represent early embryo, elongating embryo, pre-implantation embryo, post-implantation extra-embryonic membrane and fetus, respectively. Gene expression at these different time points was analyzed using our cDNA microarray. Two clustering algorithms such as *k*-means and hierarchical clustering methods identified the expression patterns of differentially expressed genes across pre-implantation period. Novel candidate genes were confirmed by real-time RT-PCR.

**Results:**

In total, 1,773 individual genes were analyzed by complete *k*-means clustering. Comparison of day 7 and day 14 revealed most genes increased during this period, and a small number of genes exhibiting altered expression decreased as gestation progressed. Clustering analysis demonstrated that trophoblast-cell-specific molecules such as placental lactogens (PLs), prolactin-related proteins (PRPs), interferon-tau, and adhesion molecules apparently all play pivotal roles in the preparation needed for implantation, since their expression was remarkably enhanced during the pre-implantation period. The hierarchical clustering analysis and RT-PCR data revealed new functional roles for certain known genes (dickkopf-1, NPM, etc) as well as novel candidate genes (AW464053, AW465434, AW462349, AW485575) related to already established trophoblast-specific genes such as PLs and PRPs.

**Conclusions:**

A large number of genes in extra-embryonic membrane increased up to implantation and these profiles provide information fundamental to an understanding of extra-embryonic membrane differentiation and development. Genes in significant expression suggest novel molecules in trophoblast differentiation.

## Background

Embryogenesis in the period from fertilization to implantation involves various morphological, cellular, and biochemical changes related to genomic activity [1]. These changes include the elongation of embryonic tissues, cell-cell contact between the mother and the embryo, and placentation. The embryo begins to form the placenta around day 20 of gestation in the bovine [2,3], while embryonic trophoblast and endometrial cells tightly unite to form placentomes on day 30 [4,5]. The bovine embryos at the blastocyst stage are 100 to 200 μm in diameter, but become 20 to 30 cm long by the time of implantation. Embryonic cells undergo both proliferation and differentiation to form the fetus and placenta throughout early embryogenesis. Reprogramming of the genome may be completed and reset during these steps, with embryonic development progressing on to temporal and spatial gene expression [6,7]. The literature data indicate that a number of specific genes are expressed during the period from blastogenesis through implantation, such as interferon-τ (IFN-τ) [8,9], matrix metalloproteinases (MMPs) [10], heparanase [11], and retinoid X receptors [12]. Vast numbers of genes change their expression levels during this period to support the complex mechanisms of embryogenesis and implantation. Although numerous molecules participate in trophoblast differentiation and placentation, the precise molecular and genetic pathways which lead to the formation of placenta remain difficult to clarify. Nevertheless, recent data from animal cloning technology where a somatic or stem cell nucleus is transplanted to an enucleated unfertilized egg, strongly suggested that inappropriate expression of genes is possibly the main cause of early embryo loss [13,14]. These studies have shown that aberrant gene expression patterns were associated with placental abnormalities in several species. Thus, it is clear that the differentiation of trophoblastic cells and the formation of placenta are tightly controlled by mechanisms which precisely govern switch on and off of the appropriate set of genes during embryogenesis. Therefore, a detailed gene expression profile in the pre-implantation embryo provides insights into the molecular mechanisms that are vital to farther our knowledge of the embryogenesis and implantation processes. Unlike traditional molecular methods that have been used to look at a single gene at one time point, a complementary DNA (cDNA) microarray is an efficient tool for analyzing tens of thousands of genes in a single experiment and to identify groups of genes that are critical during early development.

We have developed a bovine utero-placental specific cDNA microarray that contains various genes known to play key roles in extra-embryonic membrane development in the bovine [15]. To our knowledge, there has been no report to date that has evaluated the differential gene expression profile in the embryo as well as in extra-embryonic cells of the pre-implantation embryo in the bovine. Thus, we applied our cDNA microarray to detect gene expressions comprehensively during embryogenesis and implantation in the bovine. Furthermore we attempted to address the expression changes of the key genes that are involved in trophoblastic cell proliferation and differentiation. We first compared the gene expression profiles among blastocyst-stage embryos, elongated-stage embryos, and the trophoblastic tissue at the peri-implantation stage. We further characterized gene expression profiles between the trophoblastic tissue and embryo just after implantation. Comparison of expression differences of specific gene(s) provides important information on the potential role of the particular gene at specific time points of trophoblast development. Approaches such as this help identifying specific genes that are up- or down-regulated during trophoblast development.

Although there is no defined standard method that has been developed for the analysis of microarray data, application of *k*-means [16,17] and hierarchical clustering [18] methods has been shown to yield reliable data. These two methods have been successfully used to obtain information from the embryo, cytotrophoblast, and uterine tissue during the early stages of pregnancy in humans [19-22] and mice [6,23,24]. We therefore applied *k*-means and hierarchical clustering methods to identify genes involved in trophoblastic differentiation.

## Materials and Methods

### Animals and sample collection

Totally five Japanese black cows were slaughtered on Days 19, 21, 27 (two) and 28 of gestation (the day of artificial insemination was designated as Day 0), and collected embryonic membrane and fetus. Embryos were collected non-surgically on Days 7 and 14 of gestation from superovulated Japanese black cows. Namely, a follicle-stimulating hormone (FSH; Antrin-R10, Kawasaki Pharmaceutical, Kawasaki, Japan) was injected twice daily at doses of 5 mg, 3 mg, and 2 mg on the first, second, and third day of treatment, respectively. Luteolysis was induced by injecting prostaglandin F_2_α (cloprostenol 750 μg/cow; Fujita Pharmaceutical, Tokyo, Japan) on three days after the initial injection of FSH. Artificial insemination was performed both on the day of estrus (Day 0 of gestation) and the next morning, using frozen semen from Japanese black bulls. Twelve blastocysts collected on Day 7 were pooled to represent the Day 7 embryo (abbreviated as Day 7E), two expanded and elongated embryos collected on Day 14 were pooled to represent Day 14E, two embryos collected on Days 19 and 21 were used individually but designated as Day 21E (n = 2). Extra-embryonic membranes collected on Days 27 (two samples) and 28 (one sample) were used as Day 28 extra-embryonic membranes (28EEM) (n = 3), and two fetuses, excluding the extra-embryonic membranes, collected on Days 27 and 28 were used as the Day 28 fetus (28F) (n = 2). All samples were snap frozen in liquid nitrogen immediately after collection and stored at -80°C until RNA extraction. The days of collection were selected depending on the macro morphological changes of the embryos during the peri-implantation periods; bovine embryo passes into the uterus around a morula stage and develop to a blastocyst stage by Day 7. Embryo begins to elongate on Day 14 and form a long embryo of 20 to 30 cm in length on Day 20 just before implantation occurs. The embryo is easily identifiable around Day 30 around which placentome formation is visible [3,5]. All procedures for these animal experiments were carried out in accordance with the guidelines and ethics approved by the Animal Ethics Committee of the National Institute of Agrobiological Sciences for the use of animals.

### Sample RNA preparation

#### RNA extraction

The total RNA was isolated from the embryos using ISOGEN (NipponGene, Toyama, Japan) according to the manufacturer's instructions. Briefly, Samples were mixed and homogenized with ISOGEN. The aqueous phase was collected after the centrifugation. This phase was washed by chloroform for the RNA purification. The aqueous phase was collected after the centrifugation again. The total RNA pellet in the aqueous phase was obtained using the isopropanol sedimentation. Total RNA was directly used for the T7-based linear amplification for the cDNA microarray analysis. Messenger RNA (mRNA) were reverse transcribed by using the T7-oligo dT primer in the T7-based linear amplification system.

#### T7-based linear amplification

Linear-amplified antisense RNA (aRNA) was used for the cDNA microarray experiment. Because the amount of total RNA extracted was small from Day 7 blastocysts for the microarray analysis, mRNA from Day 7 blastocysts was amplified along with all other mRNA samples that were collected from Days 14 to 28 of gestation to eliminate technical variations and to efficiently compare the array data.

We used a MessageAmp aRNA kit (Ambion, Austin, TX, USA) for the T7-based linear amplification of mRNA. Synthesis and purification of cDNA and aRNA were carried out according to the manufacturer's instructions. The aRNA amplification procedure consisted of (i) first-strand cDNA synthesis (reverse transcription of mRNA), (ii) second-strand cDNA synthesis, (iii) cDNA purification, (iv) in vitro transcription, and (v) aRNA purification. The aRNA amplification for Day 7 blastocysts was done with two steps purification. The comparison between first and second round amplification was r = 0.96 (data not shown).

### cDNA microarray

#### Bovine utero-placental cDNA microarray

For the series of present experiments we used a utero-placental custom cDNA microarray developed in our laboratory, as described previously [13,15]. Briefly, a cDNA library was constructed from mRNA isolated from endometrial (caruncular and intercaruncular endometrium) and placental tissues (cotyledonary and intercotyledonary fetal membrane) of Japanese Black cows on days 0 and 10 of the estrous cycle, and days 30, 60, 100 and 245 of gestation, respectively. PCR products of about 4,000 clones from the cDNA library which was normalized by hit-picking method [15] were spotted onto glass slides robotically. Simultaneously, the clones were sequenced by using the MegaBACE 1000 DNA Sequencing System (Amersham Pharmacia Biotech, Piscataway, NJ). The array contained 3,955 spots that were clustered into 1,738 unique genes on the basis of sequence analysis. The sequenced clones were compiled and annotated by basic local alignment search tool (BLASTn), and the clone that was positioned at the top of the hierarchical tree of genes by GenBank database matching was selected. The 1,738 annotated unique genes represented 816 known genes, 530 expressed sequencing tags (ESTs) and 392 unknown novel genes. The unknown sequences were submitted to DDBJ as ESTs. The DDBJ and GenBank accession numbers of genes on this custom microarray are BP106801 to BP113049 and AB098745 to AB099150, respectively. An additional 35 genes that were not included in the cDNA library but also spotted onto the cDNA microarray were used for analysis since these genes have been shown to be characteristically expressed during gestation in bovines and humans [9,25-29].

#### cDNA microarray hybridization

We have previously reported our microarray hybridization procedures [15]. Briefly, two μg of amplified aRNA was reverse transcribed in the presence of cyanine 3 (Cy3) or Cy5 fluorescence dye (Amersham Biosciences, Buckinghamshire, UK) using SuperScript II reverse transcriptase (Invitrogen, Carlsbad, CA, USA) to make the hybridization probes. The reaction mixture was incubated for 2 hr at 42°C. The labeled probes were concentrated in a Microcon filter device (Millipore, Bendford, MA, USA), diluted in 15 μl hybridization solution (3.4 × SSC, 0.3% SDS, 20 μg poly (A), and 20 μg yeast tRNA), and applied to the microarray. Identical samples were labeled separately with either Cy3- or Cy5-dye. Thus, two hybridization reactions could be carried out with the same sample. The arrays were sequentially washed with 2 × SSC/0.5% SDS, 0.2 × SSC/0.5% SDS, and 0.2 × SSC solutions after 16 hr incubation at 65°C. The arrays were dried by centrifugation at 1,000 × g. Hybridization images were immediately scanned by a GenePix 4000B laser scanner (Axon Instrument, Union City, CA, USA) and analyzed with GenePix Pro 4.0 computer software. The data were viewed as a scatter plot between Cy3 and Cy5 intensities. Each color intensity value was individually normalized, and the average intensity value of each spot (gene) obtained from different hybridization reactions of the same sample was used for data analysis.

#### Data normalization of the cDNA microarray

Data normalization was performed by following the procedures described previously [30,31]. The local background intensity of each array spot was smoothed by local weight regression (Lowess) and subtracted from the spot intensity data. The subtracted intensity data were subjected to non-parametric regression and local variance normalization since non-parametric regression can reduce intensity-dependent biases. The accuracy is improved over that of linear regression if the points in the scatter plot of Cy3 versus (vs.) Cy5 are not distributed around a straight line. Minimum Information About a Microarray Experiment (MIAME; ) compliance was met by depositing all the data in the Gene Expression Omnibus (GEO) repository . The GEO accession numbers are as follows. Platform: GPL1221; Samples: GSM23324, GSM26510, GSM26511, GSM23327, GSM23328, GSM23329, GSM23330, GSM23331, and GSM23332; Series: GSE1414.

#### Cluster analysis of the cDNA microarray data

Data for individual genes were obtained by averaging the intensity values of analogous spots on the microarray. Data were log_2 _transformed and used for cluster analysis. The TIGR (The Institute for Genome Research) MultiExperiment Viewer (MeV) program  was used to derive the *k*-means and for hierarchical tree clustering analysis [32]. The general expression patterns of the 1,773 individual genes (including 35 manually spotted genes) were investigated by *k*-means algorithm. Data for each gene were represented by an eight-dimensional vector. *K*-means clustering was performed by division into 12 centroid centers. The distance between gene vectors was calculated by the cosine coefficient (vector angle). Hierarchical tree clustering was performed on the annotated and EST genes that demonstrated up-regulation when the comparison was made between Day 28EEM vs. Day 28F. Genes that displayed two-fold (and greater) differences were selected and the normalized expression of each sample was utilized for the cluster analysis. The distance between gene vectors was also calculated by the cosine coefficient. We adopted average linkage clustering in the hierarchical tree clustering method. Data used for the cluster analyses were as follows: Day 21E (n = 2; r = 0.84; *P *< 0.05); Day 28EEM (n = 3; r ≥ 0.90; *P *< 0.05); and Day 28F (n = 2; r = 0.90; *P *< 0.05). Other samples were used independently for cluster analysis.

### Real-time RT-PCR analysis

Gene expression profiles derived from microarray analyses were confirmed quantitatively by real-time RT-PCR analysis. The selected genes for RT-PCR included two key known genes (placental lactogen-Ala (PL-Ala) and prolactin-related protein-1 (PRP-I)) and four apparently up-regulated ESTs, and compared their expression from Day 7 through Day 28. The ESTs selected have the GenBank accession numbers AW464053, AW465434, AW462349, and AW485575.

The details of real-time RT-PCR procedures have been described in previous reports [11,33]. Briefly, fifty ng total RNA was reverse transcribed into cDNA for 30 min at 48°C by MultiScribe™ reverse transcriptase with an Oligo dT primer, dNTP mixture, MgCl_2 _and an RNase inhibitor. Primer pairs and oligonucleotide probes labeled with a reporter fluorescence dye at the 5' end and a quencher fluorescence dye at the 3' end were designed using Primer Express computer software (Applied Biosystems, Foster City, CA, USA). The primers and probes for the selected genes are listed in Table 1. The thermal cycling conditions included an initial incubation of samples at 50°C for two minutes and at 95°C for ten minutes, followed by forty cycles with each cycle at 95°C for fifteen seconds and at 60°C for one minute. The cycle threshold value (C_T_) is related to the initial quantity of the target gene in each sample determined in real time using an ABI Prism 7700 sequence detector (Applied Biosystems). The relative difference in the initial amount of each mRNA species (or cDNA) was determined by comparing the C_T _values between the samples at different stages. Bovine GAPDH was used as endogenous control. The standard curve for each gene was generated by serial dilution of plasmid containing PL-Ala, PRP-I, four ESTs, or GAPDH cDNA to quantify mRNA concentrations. A ratio of PL-Ala, PRP-I and four ESTs mRNA to GAPDH mRNA was calculated to adjust for any variation in the RT-PCR reaction. All values are presented as mean ± SD. Data were analyzed initially by ANOVA and followed by either Tukey-Kramer multiple comparison test. *P*-values of <0.05 were considered significant.

**Table 1:** Oligonucleotide primers and TaqMan probes used for real-time RT-PCR analysis

**Gene**	**Primer or TaqMan probe**	**Sequence**	**Position**
PL-Ala (J02840)	Forward	5' GCAACATTGGTGGCTAGCAA 3'	262-281
	Reverse	5' GCCCTCGCCAAACTGTTTATTA 3'	339-317
	Probe	5' CTATAGGCTCGCCAGGGAAATGTTCAATGA 3'	285-314
PRP-I (J02944)	Forward	5' CAGACAGGTTTATGAATGCCGC 3'	458-479
	Reverse	5' CGCAGGCAGTAGAACAGGTTAT 3'	541-520
	Probe	5' TCCTCTGCATCATCTAGTCACGGAGCTG 3'	483-511
AW464053	Forward	5' AATATGCCCAGGGCAAACTG 3'	296-315
	Reverse	5' TCGGGAGTTTGGAGGGAATT 3'	368-349
	Probe	5' TCAATGCCATCAAGAGCTGCCACAC 3'	323-347
AW465434	Forward	5' ACATCTCCCTGAAAGTGAACCC 3'	287-308
	Reverse	5' TCCATCCTTGCAGAAGTCTCCT 3'	369-348
	Probe	5' CCCTGGAAGCTCATCTGCAATGTAAAGC 3'	316-343
AW462349	Forward	5' GCGTGGATGGTGTCCTACTTCTA 3'	216-238
	Reverse	5' GCCACAACGAGAAACAGGAAA 3'	301-281
	Probe	5' TGTCTGTTTGCCTTTACTGGTGAGCCCT 3'	240-267
AW485575	Forward	5' CCTCTGATGAAAGATTGGGAACAG 3'	195-218
	Reverse	5' AAGTGCCAGAGATCTTGGCCT 3'	285-265
	Probe	5' TTCTCCAAACCAACCACCACCAGCTG 3'	230-255
GAPDH (U85042)	Forward	5' AAGGCCATCACCATCTTCCA 3'	178-197
	Reverse	5' CCACTACATACTCAGCACCAGCAT 3'	253-230
	Probe	5' AGCGAGATCCTGCCAACATCAAGTGG 3'	200-225

## Results

### T7-based linear amplification

We confirmed the reliability of this amplification method by comparing unamplified mRNA vs. amplified mRNA (aRNA) derived from the same bovine placental tissue after hybridization into the utero-placental cDNA microarray. We observed a significant correlation (r ≥ 0.81, *P *< 0.05; data not shown) between the unamplified mRNA and amplified aRNA. Confirmation experiments were conducted twice with reverse labeling (for a total of four times). Previous reports have also described substantial cDNA microarray population correlation between unamplified mRNA and amplified aRNA [34,35]. The correlation of our amplification was equivalent to previous studies [34,35]. Thus, amplified aRNAs can be used as representative materials in cDNA microarray experiments.

### Differential gene expression profiles determined by cluster analysis

**Figure 1:**
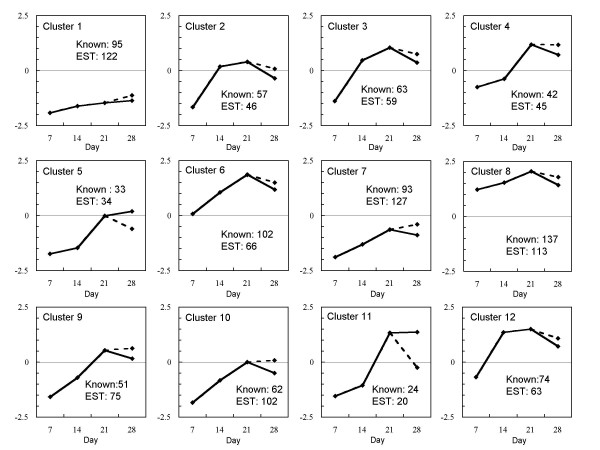
***K*-means clusters of the expression patterns of 1707 genes in a bovine embryo. **Lines refer to the *k*-means of gene expression on Days 7E to 21E, Day 28EEM and Day 28F. The solid line refers to the *k*-means center of gene expression on Days 7E to 21E and Day 28EEM. The dotted line refers to the *k*-means center of Day 21E to Day 28F.

Sixty-six genes out of a total of 1,773 were exempted from cluster analysis due to low expression intensity values. Consequently, 1,707 genes, including 833 previously annotated, were classified into 12 categories by *k*-means clustering, as illustrated in Fig. 1. Twelve *k*-means cluster profiles were summarized into three types, in which the gene-expression intensity (i) increased only from Day 7 to Day 14 and thereafter either remained constant or decreased slightly (clusters 2, 3, 12); (ii) Continued to increase until Day 21 (clusters 1, 6, 7, 8, 9, and 10); and (iii) progressively increased until Day 21 (clusters 4, 5, and 11). Clusters 1 and 7 contained more than 200 genes that included annotated and ESTs, whereas clusters 4, 5, and 11 contained fewer than 100 genes. Clusters 5 and 11 contained mostly members of the cytokine family, and clusters 4 and 5 contained ECM-related genes. In particular, trophoblast-specific genes, such as placental lactogens (PLs), prolactin-related proteins (PRPs), and pregnancy-associated glycoproteins (PAGs), were found in cluster 11.

### Temporal specific gene expression in extra-embryonic membranes

**Figure 2:**
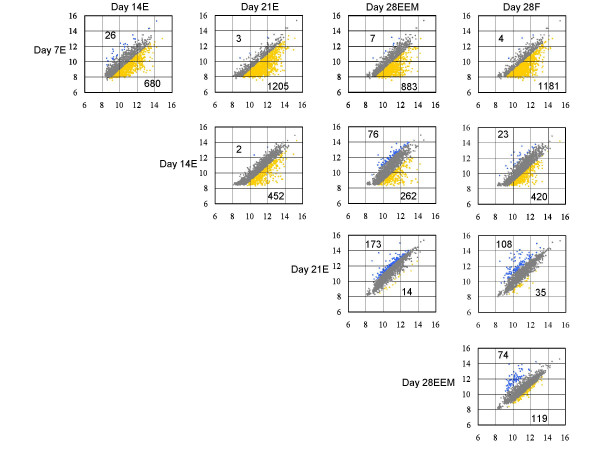
**Gene expression correlations of bovine peri-implantation embryo between Day 7E vs. Day 14E, Day 7E vs. Day 21E, Day 7E vs. Day 28EEM, Day 7E vs. Day 28F, Day 14E vs. Day 21E, Day 14E vs. Day 28EEM, Day 14E vs. Day 28F, Day 21E vs. Day 28EEM, Day 21E vs. Day 28F, and Day 28EEM vs. Day 28F. **The yellow areas highlight a greater than two-fold gene expression difference (up-regulated) between the X-axis and Y-axis samples. The blue areas highlight a greater than two-fold gene expression difference (down-regulated) between the X-axis and Y-axis samples. The gray areas highlight a 0.5- to 2-fold gene expression difference between the X-axis and Y-axis samples.

Pair-wise comparisons were made to identify genes displaying differential expression; the results are given in Fig. 2. All data considered significant exhibited an increase (up-regulation; yellow) or decrease (down-regulation; blue) of at least two-fold. Top 20 known genes of the most or less expression over two-fold differences were listed in Tables 2 to 5. All data of individual gene changes are available in an additional file on same web site of this paper or  as supplemental tables (Suppl. Tables 1–4 in Additional files – see ).

**Table 2:** Top 20 known genes of the most or less expression over two-fold differences between Day 7E vs. Day 14E – see also additional file 1

**Accession No.**	**Gene name**	**D14E/D7E**	***K*-means**	**Classification**
	**Day 14E/Day 7E down-regulated genes (<0.5)**			
L07872	Homo sapiens Jk-recombination signal binding protein	0.19	8	Apoptosis & Cell cycle
NM_001294	Homo sapiens CLPTM1	0.23	4	Cytokine family
NM_001021	Homo sapiens ribosomal protein S17 (RPS17)	0.25	8	Ribosomal
M20866	Sus scrofa cofilin	0.25	1	Cytoskeleton
M21683	Sus scrofa nonhistone protein HMG1	0.30	8	DNA binding protein
AF020508	Bos taurus PAG-6	0.36	11	Cytokine family
AF020507	Bos taurus PAG-5	0.39	11	Cytokine family
X91755	Bos taurus cathepsin L	0.42	8	Oncogene & Tumor inhibitor
NM_005722	Homo sapiens ARP2 actin-related protein 2 homolog (yeast)	0.43	8	Cytoskeleton
AF210381	Bos taurus DDVit1	0.44	4	ECM & related
AF020506	Bos taurus PAG-4	0.46	1	Cytokine family
U21660	Bos taurus phosphatidylcholine transfer protein	0.47	4	
L02897	Dog nonerythroid beta-spectrin	0.47	9	
AF166124	Homo sapiens selenoprotein X	0.49	8	
	**Day 14E/Day 7E up-regulated genes (2<)**			
X14926	Mus musculus calreticulin	24.00	12	
AB009282	Homo sapiens cytochrome b5	16.48	12	
NM_004494	Homo sapiens hepatoma-derived growth factor (HDGF)	13.52	12	Oncogene & Tumor inhibitor
AF000137	Bos taurus connective tissue growth factor precursor (CTGF)	12.18	12	Cytokine family
NM_000365	Homo sapiens triosephosphate isomerase 1 (TPI1)	10.52	12	
X89984	Homo sapiens BCL7A protein	10.31	12	Cytoskeleton
AF217197	Homo sapiens FBP interacting repressor (FIR)	9.80	12	Transcriptional regulator
NM_005720	Homo sapiens ARPC1B	9.45	3	Cytoskeleton
NM_001404	Homo sapiens EEF1G	9.43	12	Transcriptional regulator
AB003094	Bos taurus ferritin L subunit	9.38	12	
X56503	Sus scrofa casein kinase II beta subunit (CKII beta)	8.55	12	Enzyme
X13684	Bos taurus glutathione peroxidase (gpx1)	8.39	3	Enzyme
NM_002436	Homo sapiens membrane protein, palmitoylated 1 (55 kD)	8.33	12	Membrane protein
NM_005022	Homo sapiens profilin 1 (PFN1)	8.09	12	Cytoskelton
X01809	Bos taurus cathepsin 3' terminus	8.08	12	Oncogene & Tumor inhibitor
AF207664	Homo sapiens matrix metalloprotease (ADAMTS1)	8.03	3	ECM & related
X56597	Homo sapiens humFib fibrillarin	7.97	12	
AF182001	Bos taurus D4-GDP-dissociation inhibitor (D4-GDI)	7.82	3	
NM_007317	Homo sapiens kinesin-like 4 (KNSL4)	7.66	2	
M21044	Bos taurus MHC class I BoLA	7.65	2	Cytokine family

All data of individual gene changes are available in Suppl. Table 1 of the Additional file.

**Table 3:** Top 20 known genes of the most or less expression over two-fold differences between Day 14E vs. Day 21E.

**Accession No.**	**Gene name**	**D21E/D14E**	***K*-means**	**Classification**
	**Day 21E/Day 14E down-regulated genes (<0.5)**			
U21661	Rattus norvegicus myotrophin	0.28	2	Cytokine family
	**Day 21E/Day 14E up-regulated genes (2<)**			
AF004877	Homo sapiens pro-alpha 2(I) collagen (COL1A2)	16.08	4	ECM & related
U21660	Bos taurus phosphatidylcholine transfer protein	10.13	4	
X15112	Bos taurus cytochrome c oxidase subunit VIb (AED)	10.01	4	
X59504	Bos taurus prolactin-like protein (PRP-VI)	9.62	11	Cytokine family
AB008683	Bos taurus alpha2(I) collagen (COL1A2)	9.27	4	ECM & related
AF105429	Ovis aries H19	7.81	4	
X65210	Bos taurus microsatellite DNA	7.62	11	
X15975	Bos taurus PRP-V	7.59	11	Cytokine family
S74761	Bos taurus water channel protein CHIP29	7.44	9	Membrane protein
AF196320	Bos taurus Interferon-tau1C	7.39	2	Cytokine family
NM_001613	Homo sapiens actin, alpha 2 (ACTA2)	6.98	4	Cytoskeleton
L06151	Bos taurus PAG-2	6.86	11	Cytokine family
M32303	Bos taurus metalloproteinase inhibitor	6.82	11	ECM & related
NM_001553	Homo sapiens IGFBP7	6.62	9	Cytokine family
M21683	Sus scrofa nonhistone protein HMG1	6.58	8	DNA binding protein
AF020508	Bos taurus PAG-6	6.54	11	Cytokine family
AF125041	Ovis aries decorin	6.42	4	
J02944	Bos taurus PRP-1	6.40	11	Cytokine family
AB004800	Sus scrofa S100C protein	5.68	9	Cytokine family
AF192336	Bos taurus PAG-19	5.47	11	Cytokine family

All data of individual gene changes are available in Suppl. Table 2 of the Additional file.

**Table 4:** Top 20 known genes of the most or less expression over two-fold differences between Day 21E vs. Day 28EEM.

**Accession No.**	**Gene name**	**D28EEM/D21E**	***K*-means**	**Classification**
	**Day 28EEM/Day 21E down-regulated genes (<0.5)**			
AF196320	Bos taurus Interferon-tau1C	0.07	2	Cytokine family
AF033096	Avena sativa nonphototropic hypocotyl 1 (NPH1-1)	0.22	12	
L10240	Homo sapiens EMMPRIN	0.24	7	ECM & related
U21660	Bos taurus phosphatidylcholine transfer protein	0.27	4	
X15112	Bos taurus cytochrome c oxidase subunit VIb (AED)	0.28	4	
L34261	Bos taurus palmitoyl-protein thioesterase	0.29	6	Enzyme
M26576	Homo sapiens alpha-1 collagen type IV	0.29	6	ECM & related
NM_001747	Homo sapiens capping protein, gelsolin-like (CAPG)	0.32	2	Cytoskeleton
U46064	Sus scrofa aldehyde reductase (ALR1)	0.33	3	Enzyme
AF020513	Bos taurus PAG-11	0.33	12	Cytokine family
M11120	Rat 28S rRNA	0.33	2	
NM_004718	Homo sapiens COX7A2L	0.34	8	Enzyme
AF144763	Bos taurus TIMP-1	0.35	3	ECM & related
AF034607	Homo sapiens chloride channel ABP	0.36	8	Membrane protein
NM_005556	Homo sapiens keratin 7 (KRT7)	0.36	2	Cytoskeleton
AJ243656	Methanobacterium thermoautotrophicum ehbA-Q	0.37	2	
M83104	Bos taurus cytochrome b5 reductase	0.37	2	Enzyme
X59693	Bos taurus ubiquinol-cytrochrome-c reductase (subunit II)	0.38	6	Enzyme
D84557	Homo sapiens HsMcm6	0.38	12	
M77234	Homo sapiens ribosomal protein S3a	0.38	6	Ribosomal
	**Day 28EEM/Day 21E up-regulated genes (2 <)**			
AF020508	Bos taurus PAG-6	3.45	11	Cytokine family
X01912	Goat epsilon I beta-globin	3.00	9	
AF004133	Sus scrofa adipocyte membrane protein	2.66	1	Membrane protein
M73961	Ovis aries PAG-1	2.35	6	Cytokine family
J02840	Bos taurus placental lactogen (PL-Ala)	2.21	5	Cytokine family
AF192336	Bos taurus PAG-19	2.17	11	Cytokine family
AB005148	Bos taurus interleukin 1 (IL-1) receptor antagonist	2.13	1	Cytokine family
D10989	Bos taurus endothelin ETB receptor	2.12	1	
X59504	Bos taurus PRP-VI	2.08	11	Cytokine family
M80328	Bos taurus PL-Val	2.01	5	Cytokine family
Z11742	Bos taurus annexin XI	2.00	5	Apoptosis & Cell cycle

All data of individual gene changes are available in Suppl. Table 3 of the Additional file.

**Table 5:** Top 20 known genes of the most or less expression over two-fold differences between Day 28EEM vs. Day 28F.

**Accession No.**	**Gene name**	**D28F/D28EEM**	***K*-means**	**Classification**
	**Day 28F/Day 28EEM down-regulated genes (<0.5)**			
M73961	Ovis aries PAG-1	0.14	6	Cytokine family
X89984	Homo sapiens BCL7A protein	0.14	12	Cytoskeleton
X15975	Bos taurus PRP-V	0.15	11	Cytokine family
AF079545	Ovis aries placental lactogen precursor (PL)	0.16	8	Cytokine family
AF020509	Bos taurus PAG-7	0.19	11	Cytokine family
J02840	Bos taurus placental lactogen (bPL-Ala)	0.20	5	Cytokine family
X59504	Bos taurus PRP-VI	0.20	11	Cytokine family
S72871	Homo sapiens GATA-2 transcription factor	0.20	8	Transcriptional regulator
AF192336	Bos taurus PAG-19	0.22	11	Cytokine family
AF020508	Bos taurus PAG-6	0.23	11	Cytokine family
J02944	Bos taurus PRP-I	0.24	11	Cytokine family
L06151	Bos taurus PAG-2	0.25	11	Cytokine family
AF020512	Bos taurus PAG-10	0.27	5	Cytokine family
AF020514	Bos taurus PAG-12	0.28	11	Cytokine family
AF192334	Bos taurus PAG-17	0.30	11	Cytokine family
AL133034	Homo sapiens clone DKFZp727K171	0.31	11	
Z11742	Bos taurus annexin XI	0.31	5	Apoptosis & Cell cycle
U89321	Homo sapiens nucleophosmin phosphoprotein (NPM)	0.32	5	
X17614	Bos taurus 3 beta hydroxy-5-ene steroid dehydrogenase/delta 5-delta4 isomerase	0.32	5	Enzyme
AF192333	Bos taurus PAG-16	0.32	11	Cytokine family
	**Day 28F/Day 28EEM up-regulated genes (2<)**			
L34261	Bos taurus palmitoyl-protein thioesterase	4.32	6	Enzyme
AF000137	Bos taurus connective tissue growth factor precursor (CTGF)	2.85	12	Cytokine family
NM_004458	Homo sapiens FACL4 transcript variant 1	2.84	7	Enzyme
NM_006491	Homo sapiens NOVA1 transcript variant 3	2.82	1	Oncogene & Tumor inhibitor
NM_006838	Homo sapiens methionine methionyl aminopeptidase 2	2.72	4	Enzyme
AB028449	Homo sapiens helicase-MOI	2.65	9	DNA binding protein
AL080102	Homo sapiens clone DKFZp564N1916	2.64	9	
AF057300	Homo sapiens truncated RAD50 protein	2.63	3	
AB043994	Bos taurus MMP-2	2.56	7	ECM & related
NM_006719	Homo sapiens transcript variant ABLIM-m	2.53	1	Cytoskeleton
AF195417	Homo sapiens DEAD-box protein abstrakt (ABS)	2.51	2	
Z25531	Bos taurus repeat region DNA	2.41	3	
AF113682	Homo sapiens clone FLB3436 PRO0868	2.40	4	
S76474	Homo sapiens trkB	2.39	1	Cytokine family
Y16533	Ovis aries IGF-II	2.39	7	Cytokine family
M86739	Bos taurus neuropeptide Y receptor	2.38	7	Cytokine family
AF043937	Homo sapiens DHAPAT	2.37	9	Enzyme
AF144763	Bos taurus TIMP-1 protein	2.36	3	ECM & related
NM_005563	Homo sapiens stathmin 1/oncoprotein 18 (STMN1)	2.36	4	Oncogene & Tumor inhibitor
AF198487	Homo sapiens transcription factor LBP-1b	2.34	4	Transcriptional regulator

All data of individual gene changes are available in Suppl. Table 4 of the Additional file.

#### Day 7 to Day 14

A comparison between Day 7E and Day 14E clearly indicates that only 26 genes were down-regulated (14 annotated and 12 ESTs), with many genes (680) being up-regulated from Day 7 through Day 14E. The most significant genes during this period are listed in Table 2 and Suppl. Table 1 (see Additional files). They included 22 cytokine-related molecules, 44 enzymes, 21 transcriptional regulators, 16 oncogenes or tumor suppressor genes, 12 apoptosis or cell-cycle molecules, 10 heat-shock proteins (HSP), 9 cell-adhesion molecules, and 8 membrane proteins. A striking expression was found for several genes during this period, i.e. calrecticulin (X14926), which is a 55 kd calcium binding protein of the ER lumen, and caldesmon (X89984), an actin-binding protein, were two of the most significantly expressed genes. PAGs and IFN were also strongly expressed. Other cytokine molecules, such as HDGF (NM_00494), had their expression accented. Cell-function-related genes such as integrin, mucin, ADAMTS, keratin, and cytokeratin were found to be significantly increased in their expression.

#### Day 14 to Day 21

A total of 452 genes were significantly up-regulated from Day 14 through Day 21, and half of these (226 genes) were annotated by BLASTn (Fig. 2). Only two genes were down-regulated, and they contained one annotated gene, myotrophin. The expression of various PL-related genes, including PRPs, was up-regulated during this period. The expression of extra-matrix-related genes, such as collagens, proteoglycans, MMPs, extracellular MMP inducer (EMMPRIN), and heparanase, and cell adhesion molecules, such as mucin, integrins, ezrin, and certain cytokines such as insulin-like growth factors (IGFs) and epidermal growth factor receptor (EGFR), were up-regulated. IFN-τ expression was emphasized. The individual gene expression data is provided in Table 3 and Suppl. Table 2 (see Additional files).

#### Day 21 to Day 28

A comparison between Day 21E and Day 28EEM revealed that the expression of 14 genes increased slightly in Day 28EEM and 11 of these were annotated, which included PAGs, PLs, and IL-1. A total of 173 genes were significantly down-regulated in Day 28EEM, including 109 annotated genes; these genes were found mainly in *k*-means clusters 2, 3, 6, 8, and 12. The most significantly down-regulated gene during this critical period for implantation was IFN-. Down-regulation of certain MMP or matrix-related genes was also found (see Table 4 and Suppl. Table 3 in Additional files) in the top 50 genes of lowest expression.

#### Comparison between extra-embryonic membranes on Day 28 and the fetus on Day 28

**Figure 3:**
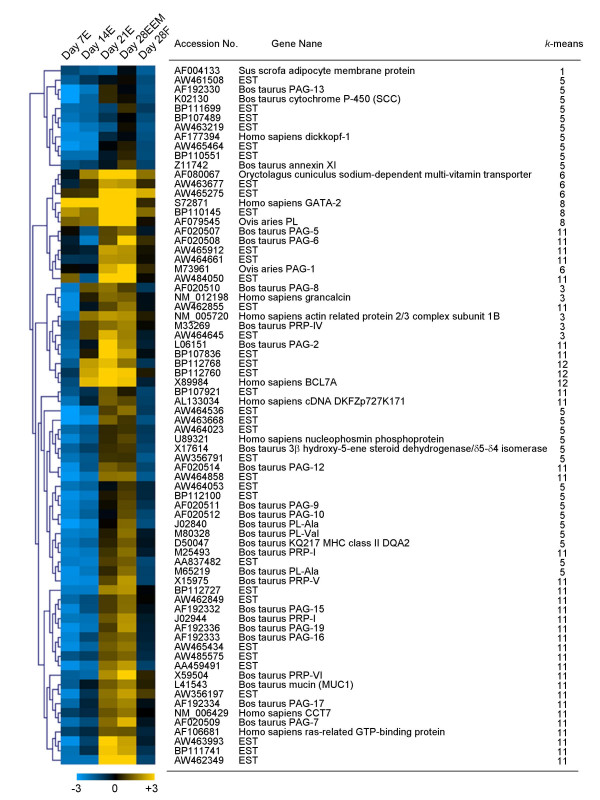
**Hierarchical tree cluster of differentially expressed genes in the bovine embryo during the implantation period (Days 7 to 28 of gestation). **Two-fold and greater differences in gene expression between Day 28EEM vs. Day 28F are selected in the expression profiles from all samples of Day 7E, 14E, 21E, 28EEM and 28F. Normalized and log_2 _transformed expression data were used for the clustering analysis. The yellow cells indicate up-regulated genes in the median of the total value; the blue cells indicate down-regulated genes in the median of the total value. The black cells indicate no changes in expression.

A total of 119 genes, including ESTs, decreased significantly in expression, with only 52 annotated genes among them in Day 28EEM (Fig. 2). However, in Day 28EEM, 74 up-regulated genes contained members of the cytokine family and molecules that play a role in cell-to-cell interactions, such as PLs, PRPs, PAGs, MHCs, and mucin, as indicated in Fig. 3. The up-regulated genes included 35 ESTs, which exhibited expression profiles similar to the cytokines during the pre-implantation period in extra-embryonic membranes. In addition to these trophoblast-specific genes, which are known to be specific to extra-embryonic membranes, other interesting genes, such as dickkopf-1, which is a Wnt signal regulation molecule, grancalcin, which is a calcium binding protein, two actin-related proteins (NM_005720, X89984), and cell proliferation related genes (NM_006429, U89321), were specifically found in extra-embryonic membranes when compared to the fetus. The actual roles of these genes in extra-embryonic cell development are as yet unknown. Individual data are provided in Table 5 and Suppl. Table 4 in Additional file 1.

### Candidate extra-embryonic membrane specific genes

**Figure 4:**
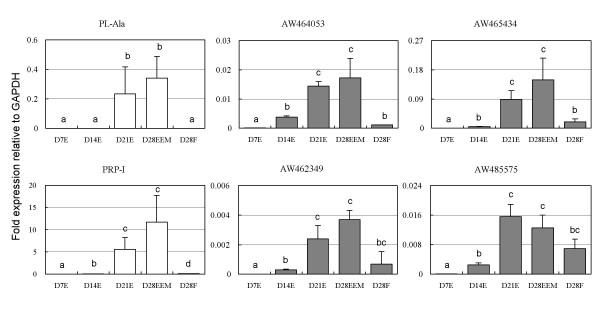
**Real-time RT-PCR analysis of PL-Ala, PRP-I, and ESTs (GenBank accession No. AW464053, AW465434, AW462349, and AW485575) mRNA in bovine embryos. **Gene expression on Days 7E to 21E, and 28EEM or 28F is provided. The expression of each mRNA was normalized to the expression of GAPDH mesured in same RNA preparation. The expression refers "means ± SD". Values with different letters are significantly different (P < 0.05).

Genes related to trophoblast cell differentiation were anticipated to change their expression when the comparison was made between Day 28EEM and Day 28F. Most of the annotated genes of the 74 genes that increased over two-fold in Day 28EEM were PL-, PRP-, and PAG-related genes. The expression of 35 ESTs showed profiles similar to those of the annotated genes during the pre-implantation period, as determined by three hierarchical analyses (Fig. 3). An attempt was made to find new genes related to extra-embryonic cell lineages. Four ESTs (AW464053, AW465434, AW462349, and AW485575) were selected and analyzed by real-time RT-PCR. The quantitative data coincided with the microarray data as shown in Figs. 3 and 4. The relative intensities of these ESTs, PL-Ala and PRP-I were similar to those of real-time RT-PCR.

## Discussion

The major reproductive wastage in farm animals is early embryo loss, i.e. the anomalous development of embryos and/or an aberration of placentation [36]. Various technologies, such as artificial insemination, embryo transfer, and cloning, have been applied to bovine reproduction [13,37]. Precise knowledge of the gene expression profile during peri-implantation is necessary to reduce early losses and to improve the reproductive efficiency of these new technologies [6,38-40]. However, little is known about the complex molecular regulation of embryos and extra-embryonic membrane development in cattle. Thus, the genes to be profiled include new, functional gene candidates. We suggest an assessment method or key gene to help clarify the complex mechanisms in early embryo and trophoblast cell proliferation and differentiation.

Various genes changes during embryonic development, particularly in extra-embryonic membranes, with specific morphological changes occur in bovine: a remarkable elongation of the embryonic membrane, fusion between fetal membrane and caruncular epithelium, endometrial reorganization [3,41,42]. Specific genes, like IFN-τ, PLs, PRPs, PAGs, IGFs, and IGFBPs have been studied as they take important roles around implantation in bovine [3,43-48]. However these molecules are essential for development of the embryo and the formation of extra-embryonic membranes, determination of their specific roles are still difficult to prove.

Microarray analyses provided the time-dependant genes profiles in accord with the progress of gestation during the peri-implantation period. Most genes that expressed from the blastocyst stage to early placentation were up-regulated, while only 14 of the identified genes exhibited down-regulation (Fig 1). For example, selenoprotein X (AF166124) exerts a stimulative function on cell proliferation, particularly in the blastocyst, since its expression decreased on Day 14 compared to Day 7. This result coincided with that in silico work [49].

We selected genes related to heat-shock proteins (HSP) to interpret comprehensive genes expression, because they were frequently found in the list of significant genes and previous data had revealed their importance in early embryo development [50]. Ten HSP-related genes out of 333 annotated genes were up-regulated over two-fold on Day 14E in comparison to Day 7E. Similar genes were detected, i.e. seven on Day 21E and three on Day 28EEM. These results suggest a specific importance for HSPs in the early development of embryos. HSPs may disturb the coordination between the conceptus and endometrium in bovines and be related to the induction of early embryo loss [51]. Aberration of HSP90 expression in mice causes a placental abnormality that is closely related to trophoblast cell differentiation and proliferation [52].

Intensive IFN-τ expression was found during the implantation period (Days 14E to 21E). The trophoblast elongates and grows into a thread-like structure during this period and produces IFN- which inhibits luteolysis and plays a role in embryonic survival by mediating embryo-maternal crosstalk [8,43,53-56]. IFN-τ not only has an anti-luteolytic function enabling the establishment of gestation in cows, but apparently also participates in various other functions necessary for embryo survival [56,57]. It has been reported that IFN-τ has an immunoregulative effect [58] and also interferon modulates the expression of MHC class I antigen in mouse trophoblast cell cultures [59]. It is interesting that MHC I and its related genes, MHC II and β-microglobulin, exhibited increased expression in the present study only during the implantation period.

Cell-adhesion molecules and cytokines, such as the integrin family, have been emphasized their importance for initiation of implantation [60-62]. Recently, Ezrin, a cytoskeletal-membrane linker molecule belonging to the ezrin-radixin-moesin (ERM) family, proved its importance for implantation in mice [63], exhibiting greater expression than in the pre-implantation stage in bovine blastocysts in the present study. The importance of adhesion molecule involvement via the adhesion-regulating molecules (ARM-1), ICAM-1, LECAM-1, Lu-ECAM-1, calcium, and integrin-binding protein (CIB) was suggested by their expression increasing around the expected implantation starting day (Day 21E).

Numerous PAG, PL, and PRP genes, involved in the differentiation of trophoblastic cell lineage, were found among the 39 annotated genes of the 74 that exhibited over a two-fold difference between extra-embryonic membranes and the fetus on Day 28. The expression of most PAG family members increased towards Day 21E. Other binucleate cell-specific genes, such as PL and PRP family members, exhibited a coordinated expression with the PAG family of genes. These results confirm previous reports from this laboratory [3,13,33,64]. PAG-5, -15, -16, -17, and -19 were distributed to *k*-means cluster 11 in the present study, whereas PAG-8 was distributed to *k*-means cluster 3 (Fig. 1). The former group of PAG family members is apparently produced by binucleate cells, while those in the latter group may be produced by other cells of the trophectoderm [46].

Microarray data suggested various new candidate genes for extra-embryonic development even they have known function. Dickkopf-1 may inhibit Wnt proteins, which influence many aspects of embryonic development [65]. Annexins are a family of structurally related calcium-dependent phospholipid binding proteins [66]. Grancalcin is a Ca (2+)-binding protein [67]. Actin-related protein 2/3 (Arp2/3) complex may be related to the actin cytoskeleton [68]. Chaperonin containing t-complex polypeptide 1 (CCT complex) is essential for the maturation of cyclin E [69]. Nucleophosmin phosphoprotein (NPM) (U89321), is a major nucleolar protein that is 20 times more abundant in tumors or proliferating cells [70]. Finally, BCL7A exhibits homology with the actin-binding protein caldesmon [71].

The most straightforward explanation for present study is that EST genes that display similar expression patterns are functionally related [72]. AW464053, AW465434, AW462349, and AW485575 gene expression patterns were similar to the expression patterns of PL and PRP-I (Figs. 3 and 4). These ESTs were submitted from the cDNA library of Soares normalized bovine placenta (Lewin et al., unpublished). The EST of AW485575 was submitted from a library made from pooled tissue of Day 20 and Day 40 bovine embryos [73]. However, the function of all four ESTs remains obscure. The AW465434 gene has a 495 bp sequence and was found to be common for 21/21 bp (e-value = 1.1) in Homo sapiens in BAC clone RP11-1246C19 (AC102953). The AW462349 gene has 545 bp sequences and was found to be common for a slight 43/47 bp (e-value = 3 × 10^-7^) in Sus scrofa clone RP44-519O7 (AC096884). The AW485575 gene has 436 bp sequences and was found to be common for a slight 157/181 bp (e-value = 6 × 10^-39^) in Homo sapiens hypothetical protein MGC39389 (BC003531). The AW464053 gene expression patterns were similar to the expression pattern of PRP-I, and the gene sequence has a portion in common with an e-value = 1 × 10^-102 ^and 4 × 10^-31 ^(common sequence = 326/371, 87% and 105/116, 90%) for PRP-VI (X59504), or an e-value = 1 × 10^-95 ^(common sequence = 351/408, 86%) for PRP-III (M27240 or NM_174160). We suggest this EST was new member of bPRP.

## Conclusions

This study provides developmental expression changes of a large number of genes in the bovine embryo during the peri-implantation period, with particular focus on genes that express in extra-embryonic membranes. The new gene candidates that were discussed here may address a new set of annotated genes and ESTs for embryonic differentiation and development. Participation of several known genes like Ezrin, CIB, Dickkopf-1, Grancalcin and EST (AW464053, AW465434 etc) in extra-embryonic membrane differentiation and development is elucidated by this microarray analysis. Fundamental investigations of this sort contribute significantly to a better understanding of the effects of various cultural conditions and cellular and genetic manipulation of embryos, including in vitro fertilization, in vitro maturation, and embryo cloning technology.

## Supplementary Material

Additional File 1 Supplement tables list the whole genes which produced the expression difference in Fig. 2 and complement Tables 2–5 beneath (see text for details). The legends of supplemental tables are as follows: Supplement Table 1; Two-fold differentially expressed genes between Day 7E vs. Day 14E. Supplement Table 2; Two-fold differentially expressed genes between Day 14E vs. Day 21E. Supplement Table 3; Two-fold differentially expressed genes between Day 21E vs. Day 28EEM. Supplement Table 4; Two-fold differentially expressed genes between Day 28EEM vs. Day 28F. Click here for file
