# Supplementary material for: cDNA microarray analysis of bovine embryo gene expression profiles during the pre-implantation period
Source: Reprod Biol Endocrinol. 2004 Nov 24;2:77. doi: 10.1186/1477-7827-2-77 (PMC535809; doi:10.1186/1477-7827-2-77)
Supplement: Additional File 1 — Supplement tables list the whole genes which produced the expression difference in Fig. 2 and complement Tables 2–5 beneath (see text for details). The legends of supplemental tables are as follows: Supplement Table 1; Two-fold differentially expressed genes between Day 7E vs. Day 14E. Supplement Table 2; Two-fold differentially expressed genes between Day 14E vs. Day 21E. Supplement Table 3; Two-fold differentially expressed genes between Day 21E vs. Day 28EEM. Supplement Table 4; Two-fold differentially expressed genes between Day 28EEM vs. Day 28F. [file 1477-7827-2-77-S1.pdf]

Supplement Table 1

Two-fold differentially expressed genes between Day7E vs Day 14E.

Accession#	Gene name	D14E/D7E	k-means	classification
	Day 14E /Day 7E down-regulated genes (< 0.5)			
AW484050	EST	0.19	11	
L07872	Homo sapiens Jk-recombination signal binding protein	0.19	8	Apoptosis & Cell cycle
NM_001294	Homo sapiens CLPTM1	0.23	4	Cytokine family
NM_001021	Homo sapiens ribosomal protein S17 (RPS17)	0.25	8	Ribosomal
M20866	Sus scrofa cofilin	0.25	1	Cytoskelton
BP110964	EST	0.26	1	
BP110413	EST	0.28	8	
M21683	Sus scrofa nonhistone protein HMG1	0.30	8	DNA binding protain
AF020508	Bos taurus PAG-6	0.36	11	Cytokine family
BP106894	EST	0.36	8	
BP110830	EST	0.38	8	
BP107823	EST	0.38	8	
AW354303	EST	0.39	8	
AF020507	Bos taurus PAG-5	0.39	11	Cytokine family
AW470389	EST	0.39	1	
X91755	Bos taurus cathepsin L	0.42	8	Oncogene & Tumor inhibitor
BP110986	EST	0.43	4	
NM_005722	Homo sapiens ARP2 actin-related protein 2 homolog (yeast)	0.43	8	Cytoskelton
AF210381	Bos taurus DDVit1	0.44	4	ECM & related
BP110238	EST	0.45	5	
AF020506	Bos taurus PAG-4	0.46	1	Cytokine family
BP110294	EST	0.47	8	
AW426990	EST	0.47	8	
U21660	Bos taurus phosphatidylcholine transfer protein	0.47	4	
L02897	Dog nonerythroid beta-spectrin	0.47	9	
AF166124	Homo sapiens selenoprotein X	0.49	8	
	Day 14E /Day 7E up-regulated genes (2 <)			
X98834	Homo sapiens zinc finger protein, Hsal2	2.00	12	DNA binding protain
AW462039	EST	2.00	8	
BP112562	EST	2.00	1	
BP111442	EST	2.00	3	
AF063243	Bos taurus ribosomal protein L30	2.01	6	Ribosomal
BP108322	EST	2.01	10	
AF192333	Bos taurus PAG-16	2.01	11	Cytokine family
AW463677	EST	2.01	6	
BP108443	EST	2.01	8	
M27239	Bos taurus PRP-II	2.02	11	Cytokine family
M12252	Chinese hamster alpha-tubulin I	2.02	8	Cytoskelton
BP106914	EST	2.02	6	
AF057300	Homo sapiens truncated RAD50 protein	2.02	3	
AW482702	EST	2.02	7	
AF109198	Bos taurus chloride channel protein p64H1	2.02	3	Membrane protein
AI617792	EST	2.02	12	
X69556	Bos taurus cpn 10	2.02	3	
AI991258	EST	2.02	7	
NM_001000	Homo sapiens ribosomal protein L39 (RPL39)	2.03	8	Ribosomal
Z25466	Bos taurus mitochondrial aspartate aminotransferase	2.03	10	Mitochondrial
BP107851	EST	2.03	7	
AF164023	Bos taurus kappa-casein (CSN3)	2.03	1	Oncogene & Tumor inhibitor
D14531	Homo sapiens homologue of rat ribosomal protein L9	2.03	6	Ribosomal
U69698	Cryptosporidium parvum heat shock protein 70(Hsp 70)	2.03	2	Heat shock protein
AF161415	Homo sapiens HSPC297	2.03	4	
NM_004738	Homo sapiens VAMP-associated protein B and C (VAPB)	2.03	2	Membrane protein
J05058	Bos taurus cytochrome c oxidase subunit IHQ (VIIb)	2.03	3	Enzyme
X04849	Bos taurus clathrin light chain a (brain LCa)	2.04	6	Membrane protein
AW483954	EST	2.04	3	
BP112917	EST	2.05	8	
M18344	Bos taurus vitamin D-dependent calcium binding protein	2.05	11	
M11341	Bos taurus low density lipoprotein receptor	2.05	10	
AI983274	EST	2.05	10	
NM_002898	Homo sapiens single stranded interacting protein 2 (RBMS2)	2.05	5	RNA binding protain
AW417894	EST	2.06	7	
AI796137	EST	2.06	7	
AC004990	Homo sapiens PAC clone RP5-1185I7 from 7q11.23-q21	2.06	10	
NM_012127	Homo sapiens Cip1-interacting zinc finger protein (CIZ1)	2.06	8	DNA binding protain
AW312756	EST	2.06	2	
BP107831	EST	2.06	3	
AW437633	EST	2.07	6	
X63678	Canis familiaris TRAM-protein	2.07	3	
BP112392	EST	2.07	10	
NM_001259	Homo sapiens cyclin-dependent kinase 6 (CDK6)	2.07	9	Apoptosis & Cell cycle
BP109609	EST	2.07	9	
X64126	Bos taurus DNA for SINE sequence Bov-1D	2.08	12	
BP110908	EST	2.08	7	
AI081391	EST	2.08	10	
NM_000661	Homo sapiens ribosomal protein L9 (RPL9)	2.08	6	Ribosomal
NM_003369	Homo sapiens UV radiation resistance associated gene	2.08	10	Apoptosis & Cell cycle
X73751	EST	2.08	7	
AW480847	EST	2.09	10	
AL036393	EST	2.09	3	
Z85980	Bos taurus capping protein, beta3 isoform	2.10	8	Cytoskelton
AW437818	EST	2.10	10	
U11927	Bos gaurus vacuolar H-ATPase subunit D (VATD)	2.10	10	Enzyme
AW464287	EST	2.10	8	
AF033095	Homo sapiens testis enhanced gene transcript protein	2.10	12	Transcriptional regulator
BP107313	EST	2.10	3	
AW344559	EST	2.10	4	
L41543	Bos taurus epithelial mucin (MUC1)	2.11	11	Cell adhesion
X63436	Bos taurus poly(A) polymerase	2.11	9	Enzyme
AF068846	Homo sapiens scaffold attachment factor A (SAF-A)	2.12	6	
AW479659	EST	2.12	6	
BP112687	EST	2.12	7	
AA112300	EST	2.12	10	
AF044670	Homo sapiens 33 kDa Vamp-associated protein (VAP33)	2.12	10	Membrane protein
X17019	Bos taurus mRNA for delta subunit of ATP synthase	2.12	8	Enzyme

L10240	Homo sapiens EMMPRIN	2.13	7	ECM & related
AW445987	EST	2.13	4	
BP109789	EST	2.13	10	
AW480521	EST	2.13	9	
BP112434	EST	2.14	8	
AL031667	Homo sapiens clone RP4-620E11	2.14	7	
AF103726	Gallus gallus peptide elongation factor 1-beta	2.14	10	Transcriptional regulator
BP112653	EST	2.14	7	
D49678	Bos taurus PAF acetylhydrolase beta-subunit	2.14	11	Enzyme
BP108071	EST	2.14	9	
AW430401	EST	2.15	7	
NM_011341	Mus musculus stromal cell derived factor 4 (Sdf4)	2.15	7	
BP107164	EST	2.15	1	
AF192334	Bos taurus PAG-17	2.15	11	Cytokine family
AI985504	EST	2.16	10	
AW359235	EST	2.16	7	
Z33879	Sus scrofa G-beta like protein	2.17	8	
AI814715	EST	2.18	2	
U17836	Bos taurus acidic ribosomal protein P2	2.18	2	Ribosomal
AW481254	EST	2.18	10	
AB032826	Bos taurus selenoprotein P	2.18	4	
AW480847	EST	2.19	7	
AW315026	EST	2.19	12	
NM_000985	Homo sapiens ribosomal protein L17 (RPL17)	2.19	6	Ribosomal
AI970619	EST	2.19	10	
AW465455	EST	2.19	9	
U37459	Mus musculus glia-derived neurotrophic growth factor	2.19	9	Cytokine family
AW260930	EST	2.20	4	
AW464570	EST	2.20	3	
AW335986	EST	2.20	6	
BP112334	EST	2.20	8	
AF144695	Mus musculus ERO1L (Ero1l)	2.20	6	
U13369	Homo sapiens ribosomal DNA complete repeating unit	2.21	7	
AF161418	Homo sapiens HSPC300	2.21	6	
AI350705	EST	2.21	10	
BP108366	EST	2.21	12	
AW466029	EST	2.22	5	
AF000152	Homo sapiens OS-4 protein	2.22	2	
AW427810	EST	2.22	10	
AW298224	EST	2.22	6	
M19962	Bos taurus cytochrome c oxidase subunit Vb (coxVb)	2.22	9	Enzyme
AA933095	EST	2.22	10	
BP109858	EST	2.23	2	
NM_006283	Homo sapiens TACC1	2.23	5	
AI061614	EST	2.23	6	
AF002693	Homo sapiens ATQL1 pseudogene	2.24	2	
BP110513	EST	2.24	9	
AW486974	EST	2.25	10	
AC002550	Homo sapiens BAC clone CIT987SK-A-101F10	2.26	9	
NM_001023	Homo sapiens ribosomal protein S20 (RPS20)	2.26	8	Ribosomal
BP112699	EST	2.26	7	
D45887	Homo sapiens calmodulin	2.26	6	Oncogene & Tumor inhibitor
BP111373	EST	2.27	10	
H44982	EST	2.27	10	
BP111869	EST	2.27	6	
BP107816	EST	2.27	10	
BP109830	EST	2.27	12	
BP112736	EST	2.27	3	
AJ004935	Urechis caupo cytoplasmic intermediate filament protein	2.27	10	Cytoskelton
BP107808	EST	2.27	9	
U65789	Bos taurus intercellular adhesion molecule-1 (bovICAM-1)	2.27	8	Cell adhesion
Z29555	Bos taurus ribosomal protein L3	2.28	8	Ribosomal
D84557	Homo sapiens HsMcm6	2.28	12	
AW461475	EST	2.28	10	
AW477773	EST	2.28	10	
BP106875	EST	2.28	6	
AF132048	Homo sapiens foocen-s	2.29	6	
NM_007278	Homo sapiens GABA(A) receptor-associated protein	2.29	9	
M22559	Bos taurus ATP synthase inhibitor protein	2.30	6	
AF081484	Homo sapiens alpha-tubulin isoform 1	2.30	6	Cytoskelton
BP111623	EST	2.30	1	
AW403893	EST	2.31	10	
NM_001015	Homo sapiens ribosomal protein S11 (RPS11)	2.31	12	Ribosomal
AW316336	EST	2.31	4	
AW307877	EST	2.31	3	
NM_005600	Homo sapiens nitrilase 1 (NIT1)	2.31	7	Enzyme
NM_006156	Homo sapiens NEDD8	2.32	12	
NM_004596	Homo sapiens small nuclear ribonucleoprotein polypeptide A	2.32	12	Nuclear protein
AW487141	EST	2.32	8	
AB028449	Homo sapiens mRNA for helicase-MOI	2.32	9	DNA binding protain
M83656	Bos taurus cytoplasmic phosphotyrosyl protein phosphatase	2.33	9	Enzyme
S61826	Homo sapiens OXPHOS system complex III mitochondrial sub	2.34	12	Mitochondrial
Z18245	Bos taurus gpolyubiquitin	2.34	12	Heat shock protein
AW249038	EST	2.35	7	
NM_000291	Homo sapiens phosphoglycerate kinase 1 (PGK1)	2.35	10	Enzyme
BP112667	EST	2.35	10	
AW484653	EST	2.35	9	
BP109619	EST	2.35	4	
BP111617	EST	2.36	10	
AW492642	EST	2.36	10	
BP109456	EST	2.36	7	
AW356092	EST	2.36	12	
AF160877	Cercopithecus aethiops ERGIC-53	2.36	1	
AF070659	Homo sapiens HSPC003	2.37	12	
Y10811	Bos taurus precystatin C	2.37	9	Oncogene & Tumor inhibitor
AF013215	Bos taurus ribosomal protein S2	2.37	12	Ribosomal
X03266	Sus scrofa inhibin beta (a)-subunit	2.37	7	Cytokine family
BP107704	EST	2.37	4	
BP112838	EST	2.38	8	
AW463993	EST	2.38	11	

AW325420	EST	2.38	10	
X03205	Homo sapiens 18S ribosomal RNA	2.39	2	
AL096857	Homo sapiens chromosome 1 which has similarities to BAT2	2.40	10	
W54378	EST	2.40	9	
AJ243486	Cyprinus carpio uncoupling protein 2	2.40	2	
AW483823	EST	2.40	10	
BP111712	EST	2.40	8	
AF062342	Homo sapiens p120 catenin isoform 4ABC (CTNND1)	2.40	10	Oncogene & Tumor inhibitor
AB032251	Homo sapiens bromodomain PHD finger transcription factor	2.41	10	Transcriptional regulator
BP113033	EST	2.41	12	
AW461681	EST	2.41	10	
AC005037	Homo sapiens BAC clone RP11-469M7 from 2	2.41	2	
AF054185	Homo sapiens proteasome subunit HSPC	2.41	8	
AJ000186	Homo sapiens MAD2 protein	2.41	7	Cytokine family
BP111440	EST	2.41	9	
AF023860	Cercopithecus aethiops cyclophilin A	2.41	6	
X76980	Bos taurus epsilon-COP	2.42	8	
AW313925	EST	2.42	10	
AW345047	EST	2.43	9	
AW476334	EST	2.43	7	
BP112666	EST	2.43	3	
AA157075	EST	2.44	1	
AF078863	Homo sapiens PTD010	2.44	8	
BP108543	EST	2.44	10	
AW345779	EST	2.44	3	
U94889	Ovis aries glyceraldehyde-3-phosphate dehydrogenase	2.44	6	Enzyme
NM_007475	Mus musculus acidic ribosomal phosphoprotein PO (Arbp)	2.45	6	Ribosomal
AI500332	EST	2.45	10	
U49973	Homo sapiens Tigger1 transposable element	2.45	9	
AW485697	EST	2.46	3	
AL133034	Homo sapiens mRNA; cDNA DKFZp727K171	2.46	11	
BP109873	EST	2.46	7	
AW314606	EST	2.47	10	
AF153686	Homo sapiens calcium binding protein precursor	2.47	3	
D63486	Homo sapiens KIAA0152	2.48	7	
NM_005872	Homo sapiens breast carcinoma amplified sequence 2	2.48	2	Oncogene & Tumor inhibitor
G29060	EST	2.49	3	
J03604	Bos taurus glutamine synthetase	2.49	9	Enzyme
U22232	Felis domesticus ribosomal protein S4	2.49	6	Ribosomal
AW417998	EST	2.49	9	
AI922214	EST	2.49	10	
M60073	Bos taurus tissue inhibitor of metalloproteinase	2.50	3	ECM & related
AV405328	EST	2.50	10	
D13891	Homo sapiens Id-2H	2.50	7	Apoptosis & Cell cycle
AF059507	Bos taurus epidermal fatty acid-binding protein (E-FABP)	2.50	9	
AF196320	Bos taurus Interferon-tau1C	2.50	2	Cytokine family
BP111789	EST	2.50	6	
AF148221	Sus scrofa Fc epsilon receptor gamma chain	2.50	3	
AW409802	EST	2.51	7	
AA933350	EST	2.51	2	
X64125	Bos taurus DNA for SINE sequence Bov-2	2.52	2	
NM_005617	Homo sapiens ERP28	2.52	8	
L06151	Bos taurus PAG-2	2.52	11	Cytokine family
S78798	Homo sapiens 1-phosphatidylinositol-4-phosphate 5-kinase isof	2.53	2	Oncogene & Tumor inhibitor
R69737	EST	2.53	10	
AI326172	EST	2.53	12	
D29805	Homo sapiens mRNA for beta-1,4-galactosyltransferase	2.54	3	Enzyme
AI439018	EST	2.54	10	
BP112742	EST	2.54	8	
BP112039	EST	2.54	7	
BP111381	EST	2.54	10	
NM_006888	Homo sapiens calmodulin 1(CALM1)	2.55	3	Oncogene & Tumor inhibitor
V01270	Rattus norvegicus 18S, 5.8S, and 28S ribosomal RNAs	2.55	3	
BP112155	EST	2.56	8	
NM_005766	Homo sapiens FERM, RhoGEF (ARHGEF)	2.56	10	Cell adhesion
AW137541	EST	2.56	7	
AW352968	EST	2.56	10	
X98054	Homo sapiens G13 protein	2.56	12	
NM_001009	Homo sapiens ribosomal protein S5 (RPS5)	2.56	3	Ribosomal
NM_002568	Homo sapiens poly(A) binding protein, cytoplasmic 1	2.57	2	DNA binding protain
AW073680	EST	2.57	7	
BP110049	EST	2.57	6	
BP111430	EST	2.57	10	
NM_009081	Mus musculus ribosomal protein L28 (Rpl28)	2.57	4	Ribosomal
AF195022	Bos taurus mucin (Muc1)	2.57	9	Cell adhesion
BP112702	EST	2.58	6	
AW113078	EST	2.58	7	
AW485507	EST	2.58	3	
AW357825	EST	2.58	10	
AA446434	EST	2.58	10	
AW461814	EST	2.60	12	
AW307679	EST	2.60	6	
AF147330	Homo sapiens clone YB24C12.	2.60	3	
L20216	Cercopithecus aethiops UV-damaged DNA-binding protein 127	2.60	3	Apoptosis & Cell cycle
NM_000991	Homo sapiens ribosomal protein L28 (RPL28)	2.60	12	Ribosomal
X69084	Bos taurus beta 2-microglobulin	2.62	6	Cytokine family
Z25531	Bos taurus repeat region DNA	2.62	3	
AA453356	EST	2.62	3	
AW345186	EST	2.62	12	
AW356197	EST	2.62	11	
BP112762	EST	2.64	10	
AW464239	EST	2.64	6	
U76713	Homo sapiens apobec-1 binding protein 1	2.64	8	
AW462171	EST	2.65	9	
AW464849	EST	2.66	10	
AW485419	EST	2.66	3	
AW312796	EST	2.66	8	
BP108284	EST	2.67	12	
AF131745	Homo sapiens clone 25110	2.67	12	
AW425519	EST	2.67	12	

AW307760	EST	2.68	2	
BP111676	EST	2.68	10	
BP108499	EST	2.69	9	
AW463647	EST	2.70	12	
AI797064	EST	2.70	2	
X74070	Homo sapiens BTF 3	2.70	3	Transcriptional regulator
AW426436	EST	2.70	12	
AI815375	EST	2.71	2	
AW461482	EST	2.71	10	
AF102850	Homo sapiens dolichyl-phosphate beta-glucosyltransferase	2.72	2	Enzyme
AW462512	EST	2.72	12	
L34015	Bos taurus cytochrome c oxidase subunit IV	2.72	12	Enzyme
BP111358	EST	2.73	10	
AW163018	EST	2.73	12	
H27490	EST	2.73	10	
AW315250	EST	2.74	10	
AW463583	EST	2.74	9	
AI353306	EST	2.74	6	
NM_006402	Homo sapiens hepatitis B virus x-interacting protein	2.75	10	Oncogene & Tumor inhibitor
AW428553	EST	2.75	12	
BP109933	EST	2.75	12	
AW315651	EST	2.76	12	
AW298937	EST	2.76	7	
AF092565	Homo sapiens splicing factor Prp8	2.76	6	Transcriptional regulator
X82879	Artificial sequences DNA for ART 2	2.76	2	
AW432050	EST	2.76	9	
J03835	Bos taurus proteolipid protein of the H+ -ATPase	2.77	8	Enzyme
BP111391	EST	2.77	10	
NM_003651	Homo sapiens cold shock domain protein A (CSDA)	2.78	8	Heat shock protein
NM_002130	Homo sapiens HMGCS1	2.78	6	Enzyme
AJ009770	Homo sapiens putative transcription factor	2.79	10	Transcriptional regulator
BP112564	EST	2.79	6	
D86562	Sus scrofa DAD1	2.79	6	Apoptosis & Cell cycle
D23660	Homo sapiens ribosomal protein	2.79	6	Ribosomal
AB003093	Bos taurus ferritin H subunit	2.79	8	
AW416500	EST	2.79	2	
M75099	Homo sapiens rapamycin- and FK506-binding protein	2.79	6	
NM_003130	Homo sapiens sorcin (SRI)	2.79	3	ECM & related
BP109321	EST	2.80	10	
AW345511	EST	2.80	9	
NM_002916	Homo sapiens replication factor C 4	2.81	12	DNA binding protain
NM_012245	Homo sapiens SKI-interacting protein (SNW1)	2.81	7	
BP111625	EST	2.82	12	
BP109513	EST	2.82	10	
AF054502	Homo sapiens clone IMAGE Consortium 302831	2.82	3	Cytokine family
U84139	Bos taurus structure-specific recognition protein 1 (SSRP1)	2.83	6	
NM_006704	Homo sapiens suppressor of G2 allele of SKP1	2.84	12	
X74728	O.cuniculus elongation factor 1 beta	2.84	6	Transcriptional regulator
AI929021	EST	2.84	9	
AB017534	Bos taurus phospholipid hydroperoxide glutathione peroxidase	2.85	9	Enzyme
X60212	Rattus norvegicus ASI	2.85	6	Ribosomal
Z33621	Bos taurus SVSP109	2.87	2	
AW479239	EST	2.87	10	
NM_002574	Homo sapiens proliferation-associated gene A	2.87	6	Oncogene & Tumor inhibitor
AW241997	EST	2.87	2	
AB015343	Homo sapiens HRIHFB2122	2.88	2	
BP109738	EST	2.88	7	
NM_002809	Homo sapiens PSMD3	2.89	12	
AW353298	EST	2.90	6	
AW354990	EST	2.90	8	
AW487044	EST	2.90	9	
AW464852	EST	2.91	9	
BP112712	EST	2.91	2	
AF057691	Mus musculus HMG box transcription factor (TCF-3)	2.91	7	Transcriptional regulator
AJ243656	Methanobacterium thermoautotrophicum ehbA-Q	2.93	2	
AW345995	EST	2.94	10	
BP111696	EST	2.94	7	
BP109363	EST	2.95	12	
BP108346	EST	2.95	7	
AF205890	Homo sapiens disabled-2	2.96	9	
AW426723	EST	2.96	12	
F22918	EST	2.98	3	
AW462829	EST	2.99	9	
AW314100	EST	3.00	10	
AW477939	EST	3.00	4	
AF172447	Mus musculus afadin	3.01	7	Cell adhesion
L32610	Homo sapiens ribonucleoprotein	3.01	7	Nuclear protein
AW415718	EST	3.01	3	
NM_003131	Homo sapiens serum response factor (SRF)	3.01	10	Transcriptional regulator
AJ132772	Bos taurus futb and rtlf	3.01	6	
AB027196	Homo sapiens RIE2 sid2705	3.02	10	
U89439	Bos taurus ubiquitin-like protein	3.02	10	Heat shock protein
AW157835	EST	3.02	2	
AW428010	EST	3.02	10	
AF143815	Bos taurus ribosomal protein (QM)	3.02	12	Ribosomal
BP107805	EST	3.03	10	
AI816672	EST	3.03	6	
X85799	Bos taurus clone TUS4	3.03	2	
BP112769	EST	3.04	7	
AI681434	EST	3.04	10	
NM_002799	Homo sapiens PSMB7	3.04	10	
AF113682	Homo sapiens clone FLB3436 PRO0868 mRNA, complete cds	3.04	4	
AW194656	EST	3.04	2	
AF094481	Homo sapiens p20-CGGBP	3.04	6	DNA binding protain
BP112290	EST	3.05	10	
Y16849	Thermobacillus xylanilyticus xynA and abfA	3.05	2	
AW358489	EST	3.06	12	
AW357737	EST	3.06	3	
AF017178	Homo sapiens pro alpha 1(I) collagen (COL1A1)	3.06	3	ECM & related
X56649	Bos taurus Annexin I	3.06	2	Apoptosis & Cell cycle

NM_001003	Homo sapiens ribosomal protein, large, P1 (RPLP1)	3.06	6	Ribosomal
D29643	Homo sapiens KIAA0115	3.07	6	
BP109528	EST	3.07	8	
U83993	Homo sapiens P2X4 purinoreceptor	3.07	2	
BP109531	EST	3.08	3	
BP111521	EST	3.08	10	
L00160	Homo sapiens phosphoglycerate kinase (pgk)	3.09	9	Enzyme
AW427611	EST	3.09	12	
NM_004500	Homo sapiens heterogeneous nuclear ribonucleoprotein C	3.10	12	
AW462084	EST	3.11	8	
AF073839	Rattus norvegicus bithoraxoid-like protein	3.11	3	
M26198	Bos taurus ASS mRNA encoding argininosuccinate synthetase	3.11	9	Enzyme
NM_010064	Mus musculus Dncic2	3.12	3	Cytoskelton
NM_000387	Homo sapiens solute carrier family 25	3.12	2	Mitochondrial
AW467629	EST	3.12	9	
M63452	Bos taurus gamma globin and globin (PSI-2) pseudogene	3.12	12	
AW358722	EST	3.13	10	
BP109463	EST	3.14	6	
U63610	Homo sapiens plectin (PLEC1)	3.15	10	Cell adhesion
AW447274	EST	3.16	12	
M15886	Bos taurus endozepine	3.16	2	
NM_006817	Homo sapiens endoplasmic reticulum lumenal protein (ERP28)	3.17	12	
AF086462	Homo sapiens clone ZD85G07	3.17	3	
AW484109	EST	3.17	12	
AW358732	EST	3.18	8	
AW336828	EST	3.18	9	
S54973	Bos taurus 20 alpha-hydroxysteroid dehydrogenase	3.18	8	Enzyme
AF043937	Homo sapiens DHAPAT	3.19	9	Enzyme
AW242801	EST	3.20	10	
AF144763	Bos taurus TIMP-1	3.21	3	ECM & related
X82321	Homo sapiens thiol-specific antioxidant	3.22	6	
X12877	Bos taurus cytokeratin A (no. 8)	3.22	12	Cytoskelton
AA165403	EST	3.23	9	
AF160639	EST	3.23	10	
AW418400	EST	3.24	12	
BP112791	EST	3.24	6	
BP108465	EST	3.24	7	
AF075377	EST	3.25	10	
AF110801	Homo sapiens interleukin-18 binding protein c precursor	3.25	9	Cytokine family
BP109473	EST	3.26	3	
BP112526	EST	3.28	8	
AW247882	EST	3.29	10	
L05093	Homo sapiens ribosomal protein L18a	3.30	6	Ribosomal
X63220	Bos taurus ubiquinone oxidoreductase complex	3.31	3	Enzyme
AW354042	EST	3.31	12	
NM_003144	Homo sapiens signal sequence receptor (SSR1)	3.31	6	
AW315092	EST	3.31	10	
D13630	Homo sapiens KIAA0005	3.31	12	
Z19087	Rattus norvegicus encoding TRAP-complex delta subunit	3.32	8	Cytokine family
AT001971	EST	3.33	2	
AW481256	EST	3.35	2	
U02610	Bos taurus acidic ribosomal protein P2	3.35	6	Ribosomal
AW409892	EST	3.35	6	
X06089	Bos taurus mitochondrial ATP synthase d-subunit	3.37	6	Mitochondrial
M58700	Sus scrofa ribonuclease inhibitor (RI)	3.37	2	
AF102243	Odocoileus hemionus DeerC106 tetranucleotide microsatellite I	3.37	2	
AI927078	EST	3.37	12	
AF083246	Homo sapiens HSPC028	3.38	12	
NM_003528	Homo sapiens H2B histone family, member Q (H2BFQ)	3.39	8	Heat shock protein
AB016798	Sus scrofa ribosomal protein L29/HIP	3.39	12	Ribosomal
BP108368	EST	3.40	2	
BP110498	EST	3.40	2	
AW344407	EST	3.40	2	
AF080067	Oryctolagus cuniculus SMVT	3.41	6	
NM_006750	Homo sapiens syntrophin beta 2 (SNTB2)	3.43	3	Cell adhesion
AF043735	Bos taurus 14-3-3 epsilon	3.43	12	
NM_000968	Homo sapiens ribosomal protein L4 (RPL4)	3.43	12	Ribosomal
BP112770	EST	3.44	3	
BP112237	EST	3.45	6	
AF086555	Homo sapiens clone ZE14E04	3.45	3	
BP109943	EST	3.46	3	
X53827	Bos taurus 7O KDa heat shock cognate protein	3.46	6	Heat shock protein
AW425551	EST	3.46	7	
NM_006320	Homo sapiens PGRMC2	3.50	3	Cytokine family
K02300	Rabbit muscle aldolase A	3.51	12	Enzyme
AW358439	EST	3.52	9	
X62048	Homo sapiens Wee1 hu	3.52	12	Apoptosis & Cell cycle
AF086313	Homo sapiens clone ZD52B10	3.53	6	
AF161464	Homo sapiens HSPC115	3.53	2	
AA933125	EST	3.55	2	
AF123303	Homo sapiens calcium-binding transporter	3.55	4	
BP109362	EST	3.56	3	
M14056	Bos taurus calpactin I heavy chain (p36) protein	3.56	8	Apoptosis & Cell cycle
X80230	Homo sapiens serine/threonine protein kinase	3.56	3	Enzyme
AW316417	EST	3.56	2	
BP108481	EST	3.57	2	
AW486434	EST	3.57	2	
M64923	Bos taurus C10 protein	3.58	12	
AW316509	EST	3.61	9	
AW464378	EST	3.63	3	
AW479092	EST	3.63	2	
AW336810	EST	3.63	12	
AW463657	EST	3.64	12	
AF161377	Homo sapiens HSPC259	3.64	6	
AW463224	EST	3.64	3	
BP109674	EST	3.65	6	
NM_000977	Homo sapiens ribosomal protein L13 (RPL13)	3.65	12	Ribosomal
X14330	Bos taurus galactose-binding lectin	3.65	12	ECM & related
BP110245	EST	3.65	8	
AW345289	EST	3.67	2	

AW487719	EST	3.67	12	
AF013213	Bos taurus elongation factor 1 alpha	3.67	8	Transcriptional regulator
AB014599	Homo sapiens KIAA0699 protein	3.67	6	
NM_001311	Homo sapiens cysteine-rich protein 1 (CRIP1)	3.68	9	
M22432	Mus musculus protein synthesis elongation factor Tu	3.69	8	Transcriptional regulator
BP111488	EST	3.69	2	
X04853	Bos taurus clathrin light chain b (lymphocyte LCb)	3.69	9	Membrane protein
AA188595	EST	3.70	12	
AW336383	EST	3.72	12	
X60195	Ovis aries 6-phosphogluconate dehydrogenase	3.73	6	Enzyme
D55670	Bos taurus thrombin inhibitor	3.75	3	
BP110942	EST	3.76	2	
AW358157	EST	3.77	10	
AF077037	Homo sapiens HSPC013	3.78	2	
AF076786	Oryctolagus cuniculus serum amyloid A-activating factor SAF-	3.78	10	Cytokine family
AW462855	EST	3.79	11	
BP111660	EST	3.80	10	
AF020513	Bos taurus PAG-11	3.82	12	Cytokine family
BP107856	EST	3.82	12	
NM_005556	Homo sapiens keratin 7 (KRT7)	3.83	2	Cytoskelton
J03275	Bos taurus ADP-ribosylation factor	3.83	6	
AA315083	EST	3.84	2	
NM_001607	Homo sapiens acetyl-Coenzyme A acyltransferase 1(ACAA1)	3.85	2	Enzyme
AW446305	EST	3.85	2	
NM_005572	Homo sapiens lamin A/C (LMNA)	3.86	3	Cytoskelton
AB017196	Sus scrofa ACY-1 and rpL29/HIP	3.87	12	Apoptosis & Cell cycle
NM_002901	Homo sapiens reticulocalbin 1, EF-hand calcium binding domai	3.90	9	
AW425870	EST	3.91	2	
AW425363	EST	3.92	10	
BP108474	EST	3.92	6	
BP110848	EST	3.93	3	
D16235	Bos taurus phospholipase C-alpha (PLC alpha)	3.94	3	Oncogene & Tumor inhibitor
AW462423	EST	3.94	6	
NM_000973	Homo sapiens ribosomal protein L8 (RPL8)	3.95	6	Ribosomal
AW356374	EST	3.96	12	
AI276594	EST	3.96	3	
AW479384	EST	3.97	2	
AW308441	EST	3.98	2	
M38520	Bos taurus cytochrome-c oxidase subunit VIa-liver isoform	4.00	8	Enzyme
AF117237	Homo sapiens prefoldin subunit 2	4.00	3	
Z48478	Bos taurus C-type natriuretic peptide	4.02	2	
AW485951	EST	4.04	12	
BP107836	EST	4.04	11	
NM_003720	Homo sapiens down syndrome critical region gene 2 (DSCR2)	4.04	3	DNA binding protain
L07919	Homo sapiens homeodomain protein DLX-2	4.05	2	Transcriptional regulator
AW465337	EST	4.05	9	
AF124819	Homo sapiens T84 colon carcinoma cell IL-1beta	4.06	2	Cytokine family
U46064	Sus scrofa aldehyde reductase (ALR1)	4.11	3	Enzyme
AF086467	Homo sapiens clone ZD86G12	4.13	12	
NM_003746	Homo sapiens dynein, cytoplasmic, light polypeptide (PIN)	4.15	12	Cytoskelton
M81129	Bos taurus superoxide dismutase	4.15	12	Enzyme
NM_002634	Homo sapiens prohibitin (PHB)	4.15	3	Oncogene & Tumor inhibitor
AW326193	EST	4.17	2	
Y09537	Sus scrofa beta-1,2-N-acetylglucosaminyltransferase II	4.19	2	Enzyme
M33269	Bos taurus PRP-IV	4.19	3	Cytokine family
BP111704	EST	4.19	6	
NM_004427	Homo sapiens early development regulator 2 (EDR2)	4.20	2	
D89060	Homo sapiens oligosaccharyltransferase	4.21	6	Enzyme
BP111331	EST	4.22	3	
NM_001960	Homo sapiens eukaryotic translation elongation factor 1 delta (I	4.22	2	Transcriptional regulator
AW416539	EST	4.23	2	
AF003998	Rattus norvegicus Golgi SNARE GS15	4.24	2	
BP107685	EST	4.27	6	
AJ010842	Homo sapiens putative ATP(GTP)-binding protein	4.29	9	
BP112697	EST	4.29	3	
BP108905	EST	4.35	1	
F22997	EST	4.37	12	
AW463719	EST	4.38	2	
NM_006705	Homo sapiens GADD45G	4.42	2	
BP112902	EST	4.43	12	
AW431699	EST	4.44	3	
AW464645	EST	4.44	3	
NM_007065	Homo sapiens CDC37 cell division cycle 37 homolog	4.45	3	Apoptosis & Cell cycle
AB015610	Chlorocebus aethiops ribosomal protein S4X	4.46	6	Ribosomal
BP108150	EST	4.46	2	
AJ249625	Paracentrotus lividus Chaperonin (hsp60)	4.48	3	Heat shock protein
NM_001101	Homo sapiens actin beta (ACTB)	4.50	12	Cytoskelton
AW382042	EST	4.50	3	
AI097036	EST	4.50	3	
AW479403	EST	4.54	12	
AL035448	EST	4.55	2	
AW462223	EST	4.55	3	
U95040	Homo sapiens transcriptional corepressor hKAP1/TIF1B	4.55	12	Transcriptional regulator
AW347614	EST	4.57	3	
AF195417	Homo sapiens DEAD-box protein abstrakt (ABS)	4.60	2	
U71273	Sus scrofa glucosidase II	4.61	6	Enzyme
AW314897	EST	4.62	12	
AW298879	EST	4.66	6	
U16336	Bos taurus cathepsin B	4.70	6	Oncogene & Tumor inhibitor
AF027200	Bos taurus ROS-GC1	4.73	6	Enzyme
AW411242	EST	4.76	3	
AW445836	EST	4.76	3	
AW483923	EST	4.76	3	
BP107868	EST	4.76	2	
AF197060	Homo sapiens HIP-55	4.77	12	
NM_003969	Homo sapiens ubiquitin-conjugating enzyme E2M (UBE2M)	4.78	2	Heat shock protein
AW427732	EST	4.81	3	
AW195100	EST	4.83	2	
M59755	Bos taurus llens aldose reductase pseudogene	4.85	6	
AF083441	Homo sapiens SUI1 isolog	4.85	12	Transcriptional regulator

BP109437	EST	4.85	9	
AW069622	EST	4.86	3	
NM_005801	Homo sapiens putative translation initiation factor (SUI1)	4.90	6	Transcriptional regulator
X51897	Rabbit histone H3.3A	4.90	3	Heat shock protein
AW461482	EST	4.91	2	
M17596	Bos taurus protein disulfide isomerase (PDI)	4.93	3	Enzyme
Z11531	Homo sapiens elongation factor-1-gamma	4.94	12	Transcriptional regulator
AW445525	EST	4.98	2	
BP112748	EST	5.04	8	
AW462183	EST	5.08	3	
NM_000979	Homo sapiens ribosomal protein L18 (RPL18)	5.11	12	Ribosomal
NM_004428	Homo sapiens ephrin-A1 (EFNA1)	5.14	3	
M16464	Bos taurus calpactin I light chain (p11)	5.15	12	Cytoskelton
AW425813	EST	5.22	2	
X72756	Bos taurus bt betaprime cop	5.22	2	
J05201	Bos taurus liver cytochrome c oxidase subunit CIX (VIII)	5.23	12	Enzyme
BP111688	EST	5.24	12	
AB017335	Homo sapiens KNSL4 and MAZ	5.30	2	Apoptosis & Cell cycle
AW462552	EST	5.31	12	
BP111710	EST	5.32	3	
AW359131	EST	5.33	3	
AW470246	EST	5.37	12	
M55409	Homo sapiens pancreatic tumor-related protein	5.43	6	Oncogene & Tumor inhibitor
BP107769	EST	5.46	3	
AF033096	Avena sativa nonphototropic hypocotyl 1 (NPH1-1)	5.47	12	
W89611	EST	5.50	2	
AF112202	Homo sapiens prenylated rab acceptor 1	5.51	3	
NM_001985	Homo sapiens electron-transfer-flavoprotein, beta polypeptide (	5.51	12	
NM_001747	Homo sapiens capping protein (actin filament), gelsolin-like (C.	5.53	2	Cytoskelton
NM_002949	Homo sapiens ribosomal protein L12 (MRPL12)	5.57	2	Ribosomal
BP111636	EST	5.69	9	
S65367	Bos taurus Ieucine aminopeptidase	5.70	2	Enzyme
NM_012198	Homo sapiens grancalcin (GCL)	5.71	3	
NM_000210	Homo sapiens integrin, alpha 6 (ITGA6)	5.74	3	Cell adhesion
S45392	Rats heat shock protein 90	5.84	6	Heat shock protein
X74191	Bos taurus microsatellite DNA INRA165	5.85	2	
U41668	Homo sapiens deoxyguanosine kinase	5.89	3	Enzyme
AW314471	EST	5.91	3	
AW482128	EST	5.94	12	
AW410172	EST	5.98	12	
NM_004393	Homo sapiens dystroglycan 1 (DAG1)	6.00	12	Cell adhesion
BP109730	EST	6.01	2	
NM_006397	Homo sapiens ribonuclease HI, large subunit (RNASEHI)	6.05	3	Enzyme
U47101	Homo sapiens NifU-like protein (hNifU)	6.12	3	
AW462425	EST	6.13	3	
L06075	Bos taurus cathepsin B	6.16	2	Oncogene & Tumor inhibitor
AF034607	Homo sapiens chloride channel ABP	6.19	8	Membrane protein
AW314471	EST	6.20	3	
AW464605	EST	6.25	12	
X04851	Bos taurus clathrin light chain a (lymphocyte LCa)	6.27	12	Membrane protein
AL049976	Homo sapiens DKFZp564B042	6.28	12	
U01946	Ovis aries secretory protein	6.29	12	
M83104	Bos taurus cytochrome b5 reductase	6.36	2	Enzyme
AW465150	EST	6.37	12	
NM_001273	Homo sapiens CHD4	6.43	12	
AW431917	EST	6.45	12	
AW464536	EST	6.52	5	
AJ000039	Bos taurus glyceraldehyde 3-phosphate dehydrogenase	6.57	12	Enzyme
AW356414	EST	6.64	3	
X04152	Bos taurus cytokeratin 19	6.67	3	Cytoskelton
NM_012193	Homo sapiens frizzled homolog 4 (Drosophila) (FZD4)	6.78	2	
D14073	Bos taurus cyclophilin B	6.78	3	
AW316336	EST	6.79	3	
BP112760	EST	6.82	12	
AW383166	EST	6.84	2	
AW482335	EST	6.87	12	
NM_011072	Mus musculus profilin 1 (Pfn1)	6.94	3	Cytoskelton
Y09206	Bos taurus MHC class 1 protein molecule D18.2	6.96	2	Cytokine family
Z24725	Homo sapiens mitogen inducible gene mig-2	6.97	3	Apoptosis & Cell cycle
U21661	Rattus norvegicus myotrophin	7.06	2	Cytokine family
AF020510	Bos taurus PAG-8	7.07	3	Cytokine family
NM_007192	Homo sapiens FACTP140	7.23	6	Transcriptional regulator
S57412	Rabbits protein phosphatase X	7.24	2	Enzyme
X16312	Homo sapiens phosvitin/casein kinase II beta subunit	7.30	12	Enzyme
AW402107	EST	7.35	3	
NM_001416	Homo sapiens EIF4A1	7.36	12	Transcriptional regulator
AF015767	Homo sapiens brain and reproductive organ-expressed protein	7.36	3	
BP112940	EST	7.45	3	
M21044	Bos taurus MHC class I BoLA	7.65	2	Cytokine family
NM_007317	Homo sapiens kinesin-like 4 (KNSL4)	7.66	2	
AW465477	EST	7.67	12	
AW427436	EST	7.76	12	
AW327928	EST	7.82	12	
AF182001	Bos taurus D4-GDP-dissociation inhibitor (D4-GDI)	7.82	3	
AW307840	EST	7.86	3	
AW465056	EST	7.88	3	
X56597	Homo sapiens humFib fibrillarin	7.97	12	
AW356736	EST	8.00	2	
AW353230	EST	8.01	3	
AF207664	Homo sapiens matrix metalloprotease (ADAMTS1)	8.03	3	ECM & related
X01809	Bos taurus cathepsin 3' terminus	8.08	12	Oncogene & Tumor inhibitor
NM_005022	Homo sapiens profilin 1 (PFN1)	8.09	12	Cytoskelton
NM_002436	Homo sapiens membrane protein, palmitoylated 1 (55kD)	8.33	12	Membrane protein
X13684	Bos taurus glutathione peroxidase (gpx1)	8.39	3	Enzyme
X56503	Sus scrofa casein kinase II beta subunit (CKII beta)	8.55	12	Enzyme
BP112768	EST	9.33	12	
AB003094	Bos taurus ferritin L subunit	9.38	12	
AW345567	EST	9.38	12	
NM_001404	Homo sapiens EEF1G	9.43	12	Transcriptional regulator
NM_005720	Homo sapiens ARPC1B	9.45	3	Cytoskelton
AW486080	EST	9.52	12	
AF217197	Homo sapiens FBP interacting repressor (FIR)	9.80	12	Transcriptional regulator
X89984	Homo sapiens BCL7A protein	10.31	12	Cytoskelton
NM_000365	Homo sapiens triosephosphate isomerase 1 (TPI1)	10.52	12	
AF000137	Bos taurus connective tissue growth factor precursor (CTGF)	12.18	12	Cytokine family
NM_004494	Homo sapiens hepatoma-derived growth factor (HDGF)	13.52	12	Oncogene & Tumor inhibitor
AB009282	Homo sapiens mRNA for cytochrome b5	16.48	12	
Z71872	EST	18.60	12	
BP111694	EST	20.40	12	
BP109950	EST	21.55	12	
X14926	Mus musculus calreticulin	24.00	12	

Supplement Table 2

Two-fold differentially expressed genes between Day 14E vs Day 21E.

Accession#	Gene name	D21E/D14I	k-means	classification
	Day 21E /Day 14E down-regulated genes (< 0.5)			
U21661	Rattus norvegicus myotrophin	0.28	2	Cytokine family
AW466068	EST	0.40	8	
	Day 21E /Day 14E up-regulated genes (2 <)			
NM_000969	Homo sapiens ribosomal protein L5 (RPL5)	2.00	6	Ribosomal
AW426758	EST	2.00	7	
AP000520	Homo sapiens chromosome 6p21.3, HLA Class I region	2.01	7	
NM_001145	Homo sapiens RNase A family 5 (ANG)	2.01	10	Enzyme
NM_004374	Homo sapiens cytochrome c oxidase subunit VIc (COX6C)	2.01	8	Mitochondrial
NM_002300	Homo sapiens lactate dehydrogenase B (LDHB)	2.01	6	Enzyme
AW461482	EST	2.01	10	
U87641	Bos taurus clone J9 mitochondrial D-loop	2.01	8	Mitochondrial
AW466029	EST	2.02	5	
AW479049	EST	2.02	7	
AW312796	EST	2.02	8	
X59693	Bos taurus ubiquinol-cytrochrome-c reductase (subunit II)	2.02	6	Enzyme
BP112760	EST	2.03	12	
X79866	Bos taurus VIa L	2.03	4	
AF161388	Homo sapiens HSPC270	2.03	8	
AW242801	EST	2.03	10	
AF225959	Mus musculus adhesion regulating molecule ARM-1	2.03	7	Cell adhesion
BP109543	EST	2.03	5	
Y10811	Bos taurus precystatin C	2.03	9	Oncogene & Tumor inhibitor
BP109609	EST	2.04	9	
BP112289	EST	2.04	7	
BP112061	EST	2.04	6	
NM_006676	Homo sapiens ubiquitin specific protease 20 (USP20)	2.05	10	Enzyme
NM_002898	Homo sapiens single stranded interacting protein 2 (RBMS2)	2.05	5	RNA binding protain
BP107876	EST	2.05	4	
AW353438	EST	2.05	7	
NM_004663	Homo sapiens RAB11A	2.05	4	Oncogene & Tumor inhibitor
AW336350	EST	2.05	7	
BP111676	EST	2.06	10	
M83656	Bos taurus cytoplasmic phosphotyrosyl protein phosphatase	2.06	9	Enzyme
BP107831	EST	2.06	3	
AF102243	Odocoileus hemionus DeerC106 tetranucleotide microsatellite I	2.06	2	
BP111076	EST	2.06	7	
NM_000978	Homo sapiens ribosomal protein L23 (RPL23)	2.06	6	Ribosomal
AW432050	EST	2.06	9	
H44982	EST	2.07	10	
NM_006704	Homo sapiens suppressor of G2 allele of SKP1	2.07	12	
AW464570	EST	2.07	3	
BP108869	EST	2.07	7	
AW465337	EST	2.07	9	
NM_012383	Homo sapiens osteoclast stimulating factor 1 (OSTF1)	2.07	11	
AJ243486	Cyprinus carpio uncoupling protein 2 (UCP2)	2.08	7	
D12816	Bos taurus actin2	2.08	5	Cytoskelton
S68400	Bos taurus elongation factor 2 3'-noncoding region homolog	2.09	6	Transcriptional regulator
X91895	Bos taurus PAS-6 and PAS-7 protein	2.09	4	ECM & related
AW463657	EST	2.09	12	
BP110521	EST	2.09	4	
BP111435	EST	2.09	10	
M63452	Bos taurus gamma globin and globin (PSI-2) pseudogene	2.10	12	
AW484739	EST	2.10	4	
AW461352	EST	2.10	5	
AW462855	EST	2.10	11	
BP108349	EST	2.10	7	
U58682	Homo sapiens ribosomal protein S28	2.10	6	Ribosomal
AW354042	EST	2.11	12	
AW403893	EST	2.11	10	
AW445836	EST	2.11	3	
BP110241	EST	2.12	6	
AW487793	EST	2.13	7	
NM_009081	Mus musculus ribosomal protein L28 (Rpl28)	2.13	4	Ribosomal
NM_004143	Homo sapiens melanocyte specific gene 1 (MSG1)	2.13	7	Cytoskelton
BP110294	EST	2.13	8	
BP108499	EST	2.14	9	
AW354303	EST	2.14	8	
AF205890	Homo sapiens disabled-2	2.14	9	
AJ000518	Bos taurus BS	2.14	8	
AW477773	EST	2.14	10	
BP106819	EST	2.14	9	
AF202922	Homo sapiens LRP16	2.14	10	
X99909	Canus sp. ribosomal protein L4	2.14	8	Ribosomal
S70448	Bos taurus F1Fo-ATP synthase complex Fo membrane domain	2.15	8	Enzyme
AF195022	Bos taurus mucin	2.15	9	Cell adhesion
NM_003011	Homo sapiens SET translocation	2.15	7	
BP108322	EST	2.15	10	
X53553	Bos taurus insulin-like growth factor II (IGF-II)	2.16	10	Cytokine family
V01270	Rattus norvegicus 18S, 5.8S, and 28S ribosomal RNAs	2.16	3	
M62428	Bos taurus polyubiquitin	2.16	7	Heat shock protein
AW069622	EST	2.17	3	
AW445452	EST	2.17	7	
AA615941	EST	2.17	10	
AW427810	EST	2.17	10	
AW314057	EST	2.18	9	
M13214	Bos taurus galactosyltransferase	2.19	7	Enzyme
U11927	Bos gaurus vacuolar H-ATPase subunit D (VATD)	2.19	10	Enzyme
U83076	EST	2.19	4	
AW130325	EST	2.19	4	
AW345491	EST	2.19	8	
BP111440	EST	2.19	9	
AF086555	Homo sapiens clone ZE14E04	2.19	3	
NM_001010	Homo sapiens ribosomal protein S6 (RPS6)	2.19	8	Ribosomal
NM_006496	Homo sapiens GNAI3	2.20	8	DNA binding protain
AW345589	EST	2.20	10	
AF181462	Bos taurus Dlk	2.21	8	

BP107512	EST	2.21	4	
X06088	Bos taurus mitochondrial ATP synthase b-subunit	2.21	8	Mitochondrial
NM_001827	Homo sapiens CDC28 protein kinase 2 (CKS2)	2.23	8	Apoptosis & Cell cycle
AW437662	EST	2.24	10	
M27239	Bos taurus PRP-II	2.24	11	Cytokine family
BP108218	EST	2.24	9	
BP107462	EST	2.25	6	
BP110830	EST	2.25	8	
BP106906	EST	2.25	8	
NM_003369	Homo sapiens UV radiation resistance associated	2.26	10	Apoptosis & Cell cycle
BP109619	EST	2.26	4	
X13698	Bos taurus ribonucleoprotein SS-B/La	2.26	10	Nuclear protein
NM_005868	Homo sapiens BET1	2.27	6	Membrane protein
AF161393	Homo sapiens HSPC275	2.27	10	
D55670	Bos taurus thrombin inhibitor	2.28	3	
NM_000291	Homo sapiens phosphoglycerate kinase 1 (PGK1)	2.28	10	Enzyme
AW464024	EST	2.28	5	
AW357737	EST	2.29	3	
BP109708	EST	2.29	9	
NM_003011	Homo sapiens SET translocation	2.29	10	
AW463583	EST	2.29	9	
NM_005572	Homo sapiens lamin A/C (LMNA)	2.29	3	Cytoskelton
BP108502	EST	2.30	9	
AW427905	EST	2.30	7	
BP110322	EST	2.30	5	
U39357	Ovis aries beta actin	2.31	6	Cytoskelton
AF016090	Bos taurus mitochondrial D-loop DNA	2.31	10	Mitochondrial
BP110722	EST	2.31	8	
AW336828	EST	2.31	9	
AW463047	EST	2.31	10	
AI936589	EST	2.32	7	
AC002550	Homo sapiens BAC clone CIT987SK-A-101F10	2.32	9	
AW463191	EST	2.32	10	
L22095	Bos taurus uterine milk protein	2.33	7	
X63436	Bos taurus poly(A) polymerase	2.33	9	Enzyme
NM_003713	Homo sapiens phosphatidic acid phosphatase type 2B	2.33	4	Enzyme
NM_001021	Homo sapiens ribosomal protein S17 (RPS17)	2.34	8	Ribosomal
AF017178	Homo sapiens pro alpha 1(I) collagen (COL1A1)	2.34	3	ECM & related
NM_002901	Homo sapiens reticulocalbin 1	2.34	9	
AF086136	Homo sapiens clone ZA89C06	2.35	7	
NM_002291	Homo sapiens laminin beta 1 (LAMB1)	2.35	10	ECM & related
NM_000995	Homo sapiens ribosomal protein L34 (RPL34)	2.35	4	Ribosomal
BP111119	EST	2.36	7	
AW425720	EST	2.36	5	
AW487044	EST	2.36	9	
AB027196	Homo sapiens RIE2 sid2705	2.37	10	
AW425637	EST	2.38	10	
AW353384	EST	2.38	4	
NM_002629	Homo sapiens phosphoglycerate mutase 1 (PGAM1)	2.38	6	
AF033096	Avena sativa nonphototropic hypocotyl 1 (NPH1-1)	2.39	12	
BP110221	EST	2.39	8	
AA945062	EST	2.39	7	
NM_003130	Homo sapiens sorcin (SRI)	2.39	3	ECM & related
AJ132583	Homo sapiens puromycin sensitive aminopeptidase	2.40	4	Enzyme
AW172770	EST	2.40	9	
AW437818	EST	2.40	10	
AF043937	Homo sapiens DHAPAT	2.40	9	Enzyme
AW357664	EST	2.41	7	
NM_008090	Mus musculus GATA binding protein 2 (Gata2)	2.41	5	Transcriptional regulator
AW417998	EST	2.42	9	
BP106875	EST	2.42	6	
M24103	Bos taurus ADP/ATP translocase T2	2.42	6	Enzyme
BP111412	EST	2.42	7	
BP110097	EST	2.42	8	
BP110223	EST	2.42	8	
AW357142	EST	2.42	5	
NM_001028	Homo sapiens ribosomal protein S25 (RPS25)	2.42	4	Ribosomal
AB018262	Homo sapiens KIAA0719 protein	2.43	4	
AW465812	EST	2.43	7	
AW486093	EST	2.44	7	
BP111373	EST	2.44	10	
AF161496	Homo sapiens HSPC147	2.44	9	
AW428010	EST	2.45	10	
AW464572	EST	2.46	4	
AC006115	Homo sapiens BAC CIT-B-470f8 (BC330812)	2.46	9	
U37459	Mus musculus glia-derived neurotrophic growth factor (GDNF)	2.47	9	Cytokine family
AW462503	EST	2.48	7	
AW477939	EST	2.48	4	
BP108543	EST	2.49	10	
NM_004530	Homo sapiens MMP-2	2.49	7	ECM & related
L41543	Bos taurus epithelial mucin (MUC1)	2.50	11	Cell adhesion
AF281160	Bos taurus heparanase	2.51	1	Cytokine family
AF020512	Bos taurus PAG-10	2.51	5	Cytokine family
M26198	Bos taurus ASS	2.51	9	Enzyme
AW314057	EST	2.51	5	
AI075188	EST	2.51	9	
AL109939	Homo sapiens clone RP3-351K20	2.52	10	
D86562	Sus scrofa DAD1	2.52	6	Apoptosis & Cell cycle
AW464053	EST	2.53	5	
AI854588	EST	2.53	1	
AW479142	EST	2.53	6	
BP107816	EST	2.53	10	
NM_005563	Homo sapiens stathmin 1/oncoprotein 18 (STMN1)	2.54	4	Oncogene & Tumor inhibitor
AW464042	EST	2.54	4	
AW462171	EST	2.55	9	
NM_001025	Homo sapiens ribosomal protein S23 (RPS23)	2.55	8	Ribosomal
AF132048	Homo sapiens foocen-s	2.55	6	
AW316163	EST	2.56	8	
BP111462	EST	2.57	4	
BP110551	EST	2.58	5	

M18344	Bos taurus vitamin D-dependent calcium binding protein	2.60	11	
AF080067	Oryctolagus cuniculus SMVT	2.60	6	
NM_001615	Homo sapiens actin gamma 2 (ACTG2)	2.60	7	Cytoskelton
U31307	EST	2.60	6	
BP110176	EST	2.61	4	
BP110206	EST	2.61	9	
AW316336	EST	2.61	4	
D17554	Homo sapiens DNA-binding protein, TAXREB107	2.61	5	DNA binding protain
BP111429	EST	2.61	5	
M19962	Bos taurus cytochrome c oxidase subunit Vb (coxVb)	2.62	9	Enzyme
AW465855	EST	2.62	10	
AW447226	EST	2.62	9	
AW465818	EST	2.62	8	
AF161415	Homo sapiens HSPC297	2.62	4	
BP107704	EST	2.63	4	
BP106856	EST	2.63	8	
BP112312	EST	2.64	9	
BP111094	EST	2.65	9	
X17614	Bos taurus 3 beta hydroxy-5-ene steroid dehydrogenase/delta 5-	2.65	5	
AW464645	EST	2.65	3	
X03444	Homo sapiens nuclear envelope protein lamin A precursor	2.67	6	Cytoskelton
AF053368	Mus musculus lysyl oxidase-related protein 2 (Lor2)	2.67	7	
AW463677	EST	2.68	6	
Z71840	EST	2.69	7	
AW345047	EST	2.69	9	
BP111379	EST	2.69	7	
NM_000210	Homo sapiens integrin alpha 6 (ITGA6)	2.70	3	Cell adhesion
NM_005766	Homo sapiens FERM, RhoGEF (ARHGEF)	2.71	10	Cell adhesion
AL117429	Homo sapiens clone DKFZp434D146	2.72	5	
AW429371	EST	2.74	7	
AF192977	Ovis aries breast basic conserved protein 1	2.74	9	
NM_005722	Homo sapiens ARP2 actin-related protein 2 (ACTR2)	2.74	8	Cytoskelton
BP112497	EST	2.74	5	
AW462223	EST	2.74	3	
AW409892	EST	2.74	6	
AW462829	EST	2.75	9	
AW347614	EST	2.76	3	
M14630	Homo sapiens prothymosin alpha	2.76	9	Oncogene & Tumor inhibitor
M11120	Rat 28S rRNA	2.77	2	
NM_000820	Homo sapiens growth arrest-specific 6 (GAS6)	2.78	7	
NM_002130	Homo sapiens 3-hydroxy-3-methylglutaryl-Coenzyme A syntha	2.79	6	Enzyme
C94642	EST	2.80	7	
AW479743	EST	2.80	9	
U22229	Felis domesticus ribosomal protein L41	2.80	8	Ribosomal
AW170467	EST	2.80	9	
U46064	Sus scrofa aldehyde reductase (ALR1)	2.80	3	Enzyme
AW207190	EST	2.80	9	
NM_006471	Homo sapiens MLCB	2.81	8	Cytoskelton
AF086462	Homo sapiens clone ZD85G07	2.83	3	
L27152	Canis familiaris tight junction protein (ZO-2)	2.83	5	Cell adhesion
AF083243	Homo sapiens HSPC025	2.84	4	
AF177394	Homo sapiens dickkopf-1 (DKK-1)	2.84	5	
AI500332	EST	2.84	10	
BP106894	EST	2.85	8	
AW463166	EST	2.86	4	
NM_007278	Homo sapiens GABA(A) receptor-associated protein	2.87	9	
NM_003155	Homo sapiens stanniocalcin (STC1)	2.87	5	
AW001992	EST	2.87	9	
AW445987	EST	2.87	4	
U43701	Homo sapiens ribosomal protein L23a	2.88	6	Ribosomal
BP110513	EST	2.88	9	
M98392	Canis familiaris oligosaccharyltransferase 48 kDa subunit	2.89	6	Enzyme
X66091	Mus musculus ASF	2.89	4	RNA binding protain
AF124728	Homo sapiens acinusS'	2.89	4	Apoptosis & Cell cycle
AL133034	Homo sapiens clone DKFZp727K171	2.91	11	
AF020513	Bos taurus PAG-11	2.91	12	Cytokine family
BP110095	EST	2.93	8	
NM_006835	Homo sapiens cyclin I (CCNI)	2.93	4	Apoptosis & Cell cycle
NM_006429	Homo sapiens CCT7	2.93	11	
NM_003144	Homo sapiens signal sequence receptor alpha (SSR1)	2.94	6	
AW463113	EST	2.94	4	
NM_004447	Homo sapiens EPS8	2.94	5	Cytokine family
AF169035	Homo sapiens protein kinase SGK3	2.94	7	Oncogene & Tumor inhibitor
M18767	Homo sapiens complement subcomponent C1s	2.95	7	
AW464852	EST	2.96	9	
AB032826	Bos taurus selp mRNA for selenoprotein P	2.96	4	
U68187	Homo sapiens extracellular matrix protein 1 alternative splice v	2.97	5	ECM & related
BP108483	EST	2.97	6	
AF106681	Homo sapiens ras-related GTP-binding protein	2.97	11	
AW480830	EST	2.97	5	
D16235	Bos taurus mRNA for phospholipase C-alpha (PLC alpha)	2.97	3	Oncogene & Tumor inhibitor
L16953	Mus musculus tumor cell dnaJ-like protein 1	2.98	8	Oncogene & Tumor inhibitor
X91755	Bos taurus cathepsin L	2.98	8	Oncogene & Tumor inhibitor
M77234	Homo sapiens ribosomal protein S3a	2.98	6	Ribosomal
AW315050	EST	2.99	5	
AF144103	Homo sapiens NJAC protein	2.99	5	
AW447662	EST	2.99	4	
BP107806	EST	2.99	7	
L39214	Ovis aries glucose transporter type 3 (GLUT-3)	3.01	8	
M17733	Homo sapiens thymosin beta-4	3.01	6	Cytoskelton
NM_006831	Homo sapiens ATP/GTP-binding protein (HEAB)	3.02	6	
D29643	Homo sapiens KIAA0115	3.03	6	
BP111810	EST	3.03	9	
J05593	Homo sapiens TIMP-2	3.03	5	ECM & related
AF192338	Bos taurus PAG-21	3.05	7	Cytokine family
X54978	Bos taurus 17,000 dalton myosin light chain	3.06	4	Cytoskelton
D30750	Bos taurus Msx-1	3.06	10	Transcriptional regulator
AF210381	Bos taurus DDVit1	3.06	4	ECM & related
AF123303	Homo sapiens calcium-binding transporter	3.08	4	
D23660	Homo sapiens ribosomal protein	3.09	6	Ribosomal

AF092535	Homo sapiens mitogen-activated protein kinase p38delta	3.09	7	Cytokine family
L10240	Homo sapiens EMMPRIN	3.10	7	ECM & related
BP108456	EST	3.11	5	
M98498	Bos taurus ezrin	3.11	6	Cell adhesion
AW482909	EST	3.11	4	
X12796	Bos taurus high mobility group 1 (HMG1) protein	3.13	7	Transcriptional regulator
BP113042	EST	3.13	5	
AW464023	EST	3.14	5	
AW402107	EST	3.14	3	
AL137647	Homo sapiens clone DKFZp586M2424	3.14	10	
BP107808	EST	3.15	9	
AI985504	EST	3.17	10	
AB043994	Bos taurus MMP-2	3.19	7	ECM & related
AF096304	Homo sapiens putative sterol reductase SR-1 (TM7SF2)	3.22	10	Oncogene & Tumor inhibitor
AB028449	Homo sapiens helicase-MOI	3.24	9	DNA binding protain
AF192334	Bos taurus PAG-17	3.26	11	Cytokine family
AW173315	EST	3.30	10	
NM_005556	Homo sapiens keratin 7 (KRT7)	3.31	2	Cytoskelton
AF020511	Bos taurus PAG-9	3.31	5	Cytokine family
AW485227	EST	3.33	10	
AF133425	Homo sapiens tetraspanin TM4-C	3.34	9	
BP107921	EST	3.34	11	
AW446540	EST	3.35	9	
BP107862	EST	3.36	4	
BP110726	EST	3.37	9	
AW462037	EST	3.38	5	
NM_000178	Homo sapiens glutathione synthetase (GSS)	3.39	10	Enzyme
NM_005731	Homo sapiens actin related protein 2/3 complex, subunit 2	3.42	6	Cytoskelton
BP108275	EST	3.42	5	
NM_001425	Homo sapiens epithelial membrane protein 3 (EMP3)	3.43	10	Membrane protein
Y16698	Homo sapiens HIV associated non-Hodgkin's lymphoma	3.44	5	Oncogene & Tumor inhibitor
AW485575	EST	3.44	11	
AW462849	EST	3.45	11	
U89321	Homo sapiens nucleophosmin phosphoprotein (NPM)	3.45	5	
BP107341	EST	3.48	9	
X59617	Homo sapiens large subunit ribonucleotide reductase (RR1)	3.53	4	Enzyme
AW425334	EST	3.55	4	
NM_006838	Homo sapiens methionine methionyl aminopeptidase 2	3.56	4	Enzyme
AF192332	Bos taurus PAG-15	3.59	11	Cytokine family
M26576	Homo sapiens alpha-1 collagen type IV	3.59	6	ECM & related
AW358439	EST	3.60	9	
BP106942	EST	3.60	6	
BP110199	EST	3.61	4	
D50047	Bos taurus KQ217 MHC class II DQA2	3.62	5	Cytokine family
NM_003922	Homo sapiens hect domain and RCC1-like domain 1	3.62	10	
BP112988	EST	3.62	9	
BP107252	EST	3.66	4	
AW417930	EST	3.66	9	
AF210379	Bos taurus matrix Gla protein	3.68	5	ECM & related
AW356791	EST	3.68	5	
AW418429	EST	3.68	9	
AA157933	EST	3.69	9	
BP110145	EST	3.74	8	
BP112105	EST	3.74	9	
AW337037	EST	3.75	10	
L07872	Homo sapiens Jk-recombination signal binding protein	3.75	8	Apoptosis & Cell cycle
M73962	Bos taurus PAG-1	3.79	5	Cytokine family
BP111196	EST	3.80	9	
BP107825	EST	3.82	4	
L34261	Bos taurus palmitoyl-protein thioesterase	3.85	6	Enzyme
AW326216	EST	3.87	6	
AF192333	Bos taurus PAG-16	3.89	11	Cytokine family
K02130	Bos taurus cytochrome P-450 (SCC)	3.90	5	
AB004272	Bos taurus Placenta Growth Factor (PLGF)	3.91	5	Cytokine family
AL080102	Homo sapiens clone DKFZp564N1916	3.92	9	
AI887421	EST	3.94	4	
AW311764	EST	4.00	6	
AW465464	EST	4.01	5	
AW344418	EST	4.02	9	
AW430319	EST	4.03	6	
U49973	Homo sapiens Tigger1 transposable element	4.05	9	
AV404300	EST	4.08	7	
NM_001294	Homo sapiens CLPTM1	4.11	4	Cytokine family
AF020507	Bos taurus PAG-5	4.11	11	Cytokine family
J02840	Bos taurus placental lactogen (bPL-Ala)	4.14	5	Cytokine family
AW356197	EST	4.16	11	
Y00712	Bos taurus proteoglycan II	4.20	9	ECM & related
BP106981	EST	4.20	6	
NM_001006	Homo sapiens ribosomal protein S3A (RPS3A)	4.24	6	Ribosomal
AW482131	EST	4.25	4	
V00493	Homo sapiens alpha globin	4.26	9	
AI052255	EST	4.26	9	
U02892	Bos taurus Angus 70 kda heat shock protein-2 (HSP70-2)	4.28	6	Heat shock protein
D90069	Bos taurus adenylate kinase isozyme 2	4.29	9	Oncogene & Tumor inhibitor
BP112100	EST	4.30	5	
M80328	Bos taurus PL-Val	4.32	5	Cytokine family
J03604	Bos taurus glutamine synthetase	4.38	9	Enzyme
L47641	Bos taurus type III pro-collagen	4.39	4	ECM & related
D88033	Bos taurus elenoprotein P-like protein	4.43	9	
AF144628	Mus musculus SLIT2	4.47	9	
NM_003289	Homo sapiens tropomyosin 2 (beta) (TPM2)	4.47	11	Cytoskelton
M73961	Ovis aries PAG-1	4.52	6	Cytokine family
BP106989	EST	4.52	8	
BP106952	EST	4.53	6	
AI081391	EST	4.66	10	
BP106971	EST	4.67	8	
AW464827	EST	4.75	4	
AF153062	Canis familiaris type I collagen pro-alpha1(I) chain (COL1A1)	4.76	4	ECM & related
M65219	Bos taurus placental lactogen (PL-Ala)	4.76	5	Cytokine family
AF059507	Bos taurus epidermal fatty acid-binding protein (E-FABP)	4.80	9	

J05391	Bos taurus glycoprotein III (GpIII)	4.82	5	ECM & related
AF192330	Bos taurus PAG-13	4.84	5	Cytokine family
U09861	Bos taurus 70 kDA heat-shock protein (hsp70)	4.86	4	Heat shock protein
AF079545	Ovis aries placental lactogen precursor (PL)	4.90	8	Cytokine family
AC005041	Homo sapiens BAC clone RP11-523H20	4.95	10	
AW417073	EST	4.95	9	
L02897	Dog nonerythroid beta-spectrin	5.01	9	
AW481108	EST	5.05	4	
AF020514	Bos taurus PAG-12	5.06	11	Cytokine family
AW347854	EST	5.08	6	
AF020509	Bos taurus PAG-7	5.14	11	Cytokine family
AW465912	EST	5.20	11	
M25493	Bos taurus PRP-I	5.22	11	Cytokine family
AW464661	EST	5.29	11	
AF192336	Bos taurus PAG-19	5.47	11	Cytokine family
AW260930	EST	5.60	4	
AI299947	EST	5.67	6	
AB004800	Sus scrofa S100C protein	5.68	9	Cytokine family
AW464536	EST	5.78	5	
BP111926	EST	5.83	11	
AW465434	EST	5.86	11	
AW465275	EST	6.23	6	
AA837482	EST	6.27	5	
J02944	Bos taurus PRP-1	6.40	11	Cytokine family
AF125041	Ovis aries decorin	6.42	4	
AF020508	Bos taurus PAG-6	6.54	11	Cytokine family
M21683	Sus scrofa nonhistone protein HMG1	6.58	8	DNA binding protain
NM_001553	Homo sapiens IGFBP7	6.62	9	Cytokine family
BP110986	EST	6.62	4	
M32303	Bos taurus metalloproteinase inhibitor	6.82	11	ECM & related
L06151	Bos taurus PAG-2	6.86	11	Cytokine family
NM_001613	Homo sapiens actin, alpha 2 (ACTA2)	6.98	4	Cytoskelton
AW411362	EST	7.24	9	
AF196320	Bos taurus Interferon-tau1C	7.39	2	Cytokine family
S74761	Bos taurus water channel protein CHIP29	7.44	9	Membrane protein
X15975	Bos taurus PRP-V	7.59	11	Cytokine family
X65210	Bos taurus microsatellite DNA	7.62	11	
AF105429	Ovis aries H19	7.81	4	
AA459491	EST	8.03	11	
BP111741	EST	8.28	11	
BP107836	EST	8.34	11	
AW461617	EST	8.75	10	
AW464858	EST	8.86	11	
AB008683	Bos taurus alpha2(I) collagen (COL1A2)	9.27	4	ECM & related
AW463668	EST	9.53	5	
X59504	Bos taurus prolactin-like protein (PRP-VI)	9.62	11	Cytokine family
X15112	Bos taurus cytochrome c oxidase subunit VIb (AED)	10.01	4	
AW447630	EST	10.10	4	
U21660	Bos taurus phosphatidylcholine transfer protein	10.13	4	
AW484058	EST	10.27	9	
BP112727	EST	10.74	11	
BP107329	EST	11.33	4	
BP111398	EST	12.04	11	
AW463993	EST	12.50	11	
AW486937	EST	14.86	4	
AF004877	Homo sapiens pro-alpha 2(I) collagen (COL1A2)	16.08	4	ECM & related
AW462349	EST	19.49	11	
AW484050	EST	20.80	11	
BP108333	EST	22.13	11	

Supplement Table 3

Two-fold differentially expressed genes between Day 21E vs Day 28EEM.

Accession#	Gene name	D28M/D21E	k-means	classification
	Day 28EEM /Day 21E down-regulated genes (< 0.5)			
BP108333	EST	0.05	11	
AF196320	Bos taurus Interferon-tau1C	0.07	2	Cytokine family
AI299947	EST	0.12	6	
BP107329	EST	0.15	4	
AW486937	EST	0.15	4	
BP111398	EST	0.20	11	
AF033096	Avena sativa nonphototropic hypocotyl 1 (NPH1-1)	0.22	12	
AW461617	EST	0.23	10	
L10240	Homo sapiens EMMPRIN	0.24	7	ECM & related
U21660	Bos taurus phosphatidylcholine transfer protein	0.27	4	
AW347854	EST	0.27	6	
X15112	Bos taurus cytochrome c oxidase subunit VIb (AED)	0.28	4	
L34261	Bos taurus palmitoyl-protein thioesterase	0.29	6	Enzyme
M26576	Homo sapiens alpha-1 collagen type IV	0.29	6	ECM & related
NM_001747	Homo sapiens capping protein, gelsolin-like (CAPG)	0.32	2	Cytoskelton
BP109933	EST	0.33	12	
U46064	Sus scrofa aldehyde reductase (ALR1)	0.33	3	Enzyme
AF020513	Bos taurus PAG-11	0.33	12	Cytokine family
M11120	Rat 28S rRNA	0.33	2	
AW479517	EST	0.34	8	
NM_004718	Homo sapiens COX7A2L	0.34	8	Enzyme
BP112770	EST	0.35	3	
AI985504	EST	0.35	10	
AF144763	Bos taurus TIMP-1	0.35	3	ECM & related
AF034607	Homo sapiens chloride channel ABP	0.36	8	Membrane protein
NM_005556	Homo sapiens keratin 7 (KRT7)	0.36	2	Cytoskelton
AJ243656	Methanobacterium thermoautotrophicum ehbA-Q	0.37	2	
M83104	Bos taurus cytochrome b5 reductase	0.37	2	Enzyme
BP107836	EST	0.38	11	
AW427611	EST	0.38	12	
X59693	Bos taurus ubiquinol-cytrochrome-c reductase (subunit II)	0.38	6	Enzyme
D84557	Homo sapiens HsMcm6	0.38	12	
M77234	Homo sapiens ribosomal protein S3a	0.38	6	Ribosomal
AF033095	Homo sapiens testis enhanced gene transcript protein	0.38	12	Transcriptional regulator
AW482335	EST	0.38	12	
AI500332	EST	0.38	10	
AB043994	Bos taurus MMP-2	0.39	7	ECM & related
NM_003720	Homo sapiens down syndrome critical region 2 (DSCR2)	0.39	3	DNA binding protain
AV404300	EST	0.39	7	
AI827165	EST	0.39	8	
AB009282	Homo sapiens mRNA for cytochrome b5	0.39	12	
NM_002949	Homo sapiens ribosomal protein L12 (MRPL12)	0.39	2	Ribosomal
D86562	Sus scrofa DAD1	0.39	6	Apoptosis & Cell cycle
U49973	Homo sapiens Tigger1 transposable element	0.39	9	
AF086462	Homo sapiens clone ZD85G07	0.39	3	
BP111625	EST	0.40	12	
AJ000039	Bos taurus glyceraldehyde 3-phosphate dehydrogenase	0.40	12	Enzyme
AF197060	Homo sapiens src homology 3 domain-containing protein HIP-[illegible]	0.40	12	
AW463657	EST	0.40	12	
AW484367	EST	0.40	8	
AF083246	Homo sapiens HSPC028	0.40	12	
AF160639	EST	0.40	10	
J03604	Bos taurus glutamine synthetase	0.40	9	Enzyme
AW464827	EST	0.40	4	
NM_002574	Homo sapiens proliferation-associated gene A	0.40	6	Oncogene & Tumor inhibitor
AJ249625	Paracentrotus lividus Chaperonin (hsp60)	0.41	3	Heat shock protein
Z25531	Bos taurus repeat region DNA	0.41	3	
NM_000365	Homo sapiens triosephosphate isomerase 1 (TPI1)	0.41	12	
AW416500	EST	0.41	2	
NM_002130	Homo sapiens HMGCS1	0.41	6	Enzyme
AC002550	Homo sapiens Chromosome 16 BAC clone CIT987SK-A-101F	0.41	9	
AI326172	EST	0.41	12	
NM_002107	Homo sapiens H3 histone, family 3A (H3F3A)	0.42	12	Heat shock protein
AW485507	EST	0.42	3	
NM_001827	Homo sapiens CDC28 protein kinase 2 (CKS2)	0.42	8	Apoptosis & Cell cycle
NM_004393	Homo sapiens dystroglycan 1 (DAG1)	0.42	12	Cell adhesion
AF195417	Homo sapiens DEAD-box protein abstrakt (ABS)	0.42	2	
AW445987	EST	0.42	4	
AF083441	Homo sapiens SUI1 isolog	0.42	12	Transcriptional regulator
D14074	Bos taurus cyclophilin	0.42	8	
AF002693	Homo sapiens ATQL1 pseudogene	0.42	2	
M16464	Bos taurus calpactin I light chain (p11)	0.42	12	Cytoskelton
X16312	Homo sapiens phosvitin/casein kinase II beta subunit	0.43	12	Enzyme
AC005041	Homo sapiens BAC clone RP11-523H20 from Ch2	0.43	10	
AB003094	Bos taurus ferritin L subunit	0.43	12	
NM_005801	Homo sapiens putative translation initiation factor (SUI1)	0.43	6	Transcriptional regulator
NM_004596	Homo sapiens small nuclear ribonucleoprotein polypeptide A	0.43	12	Nuclear protein
M55409	Homo sapiens pancreatic tumor-related protein	0.43	6	Oncogene & Tumor inhibitor
AW195100	EST	0.43	2	
X76980	Bos taurus epsilon-COP	0.43	8	
U31307	EST	0.43	6	
AW353298	EST	0.43	6	
AW485951	EST	0.43	12	
AF131745	Homo sapiens clone 25110	0.43	12	
Y16849	Thermobacillus xylanilyticus xynA and abfA	0.43	2	
AW298224	EST	0.44	6	
NM_006704	Homo sapiens suppressor of G2 allele of SKP1	0.44	12	
J05201	Bos taurus liver cytochrome c oxidase subunit CIX (VIII)	0.44	12	Enzyme
AF094481	Homo sapiens CGGBP	0.44	6	DNA binding protain
NM_000387	Homo sapiens solute carrier family 25	0.44	2	Mitochondrial
AW462084	EST	0.44	8	
AW486434	EST	0.44	2	
AA933350	EST	0.45	2	
NM_006496	Homo sapiens GNAI3	0.45	8	DNA binding protain
AF078863	Homo sapiens PTD010	0.45	8	

BP109730	EST	0.45	2	
X12877	Bos taurus cytokeratin A (no. 8)	0.45	12	Cytoskelton
AF015767	Homo sapiens brain and reproductive organ-expressed protein	0.45	3	
AW359222	EST	0.46	1	
AW359131	EST	0.46	3	
U09861	Bos taurus 70 kDA heat-shock protein (hsp70)	0.46	4	Heat shock protein
NM_005572	Homo sapiens lamin A/C (LMNA)	0.46	3	Cytoskelton
AW431917	EST	0.46	12	
M24103	Bos taurus ADP/ATP translocase T2	0.46	6	Enzyme
AW157835	EST	0.46	2	
Z25466	Bos taurus mitochondrial aspartate aminotransferase	0.46	10	Mitochondrial
NM_010064	Mus musculus Dncic2	0.46	3	Cytoskelton
NM_001416	Homo sapiens EIF4A1	0.46	12	Transcriptional regulator
BP112666	EST	0.46	3	
V01270	Rattus norvegicus 18S, 5.8S, and 28S ribosomal RNAs	0.46	3	
NM_002300	Homo sapiens lactate dehydrogenase B (LDHB)	0.46	6	Enzyme
X56503	Sus scrofa casein kinase II beta subunit (CKII beta)	0.46	12	Enzyme
V00654	Bos taurus mitochondrial genome	0.46	8	Mitochondrial
Z11531	Homo sapiens elongation factor-1-gamma	0.46	12	Transcriptional regulator
U01946	Ovis aries secretory protein	0.46	12	
M83656	Bos taurus cytoplasmic phosphotyrosyl protein phosphatase	0.46	9	Enzyme
NM_010447	Mus musculus heterogeneous nuclear ribonucleoprotein A1	0.46	6	Nuclear protein
AA188595	EST	0.46	12	
Z33879	Sus scrofa G-beta like protein	0.47	8	
AF044670	Homo sapiens 33 kDa Vamp-associated protein (VAP33)	0.47	10	Membrane protein
AF102850	Homo sapiens dolichyl-phosphate beta-glucosyltransferase	0.47	2	Enzyme
X56597	Homo sapiens humFib fibrillarin	0.47	12	
X82879	Artificial sequences DNA for ART 2	0.47	2	
U21661	Rattus norvegicus myotrophin	0.47	2	Cytokine family
U65789	Bos taurus intercellular adhesion molecule-1 (ICAM-1)	0.47	8	Cell adhesion
X65210	Bos taurus microsatellite DNA	0.47	11	
AW336383	EST	0.47	12	
X06088	Bos taurus mRNA for mitochondrial ATP synthase b-subunit	0.47	8	Mitochondrial
AF161415	Homo sapiens HSPC297	0.47	4	
AW427732	EST	0.47	3	
AW464287	EST	0.48	8	
AA933125	EST	0.48	2	
AW466068	EST	0.48	8	
D90069	Bos taurus adenylate kinase isozyme 2	0.48	9	Oncogene & Tumor inhibitor
AW327109	EST	0.48	8	
BP112940	EST	0.48	3	
X02493	Homo sapiens interferon-inducible (cDNA 6-26)	0.48	6	Cytokine family
AW465818	EST	0.48	8	
M38520	Bos taurus cytochrome-c oxidase subunit VIa-liver isoform	0.48	8	Enzyme
X69084	Bos taurus beta 2-microglobulin	0.48	6	Cytokine family
AW356038	EST	0.48	8	
NM_001294	Homo sapiens CLPTM1	0.48	4	Cytokine family
BP106883	EST	0.49	8	
NM_003528	Homo sapiens H2B histone family, member Q (H2BFQ)	0.49	8	Heat shock protein
NM_001404	Homo sapiens eukaryotic translation elongation factor 1 gamma	0.49	12	Transcriptional regulator
AF092565	Homo sapiens splicing factor Prp8	0.49	6	Transcriptional regulator
U84139	Bos taurus structure-specific recognition protein 1 (SSRP1)	0.49	6	
BP111975	EST	0.49	8	
BP112742	EST	0.49	8	
NM_002809	Homo sapiens proteasome 26S subunit, non-ATPase, 3 (PSMD:	0.49	12	
AW432050	EST	0.49	9	
U17836	Bos taurus acidic ribosomal protein P2	0.49	2	Ribosomal
AW463047	EST	0.49	10	
AF102243	Odocoileus hemionus DeerC106 tetranucleotide microsatellite I	0.49	2	
AB006852	Rattus norvegicus phosphoarginine phosphatase	0.49	3	Enzyme
NM_006838	Homo sapiens methionine methionyl aminopeptidase 2	0.49	4	Enzyme
AW464605	EST	0.49	12	
AI908445	EST	0.50	3	
U76713	Homo sapiens apobec-1 binding protein 1	0.50	8	
AW461814	EST	0.50	12	
NM_006082	Homo sapiens tubulin, alpha, ubiquitous (K-ALPHA-1)	0.50	8	Cytoskelton
AW470246	EST	0.50	12	
M59755	Bos taurus llens aldose reductase pseudogene	0.50	6	
U71273	Sus scrofa glucosidase II	0.50	6	Enzyme
D16235	Bos taurus phospholipase C-alpha (PLC alpha)	0.50	3	Oncogene & Tumor inhibitor
AT001971	EST	0.50	2	
AI276594	EST	0.50	3	
NM_003969	Homo sapiens ubiquitin-conjugating enzyme E2M (UBE2M)	0.50	2	Heat shock protein
BP107694	EST	0.50	1	
AW307760	EST	0.50	2	
BP111504	EST	0.50	8	
AB000425	Sus scrofa endopeptidase 24.16	0.50	8	Oncogene & Tumor inhibitor
	Day 28EEM /Day 21E up-regulated genes (2 <)			
Z11742	Bos taurus annexin XI	2.00	5	Apoptosis & Cell cycle
M80328	Bos taurus PL-Val	2.01	5	Cytokine family
X59504	Bos taurus PRP-VI	2.08	11	Cytokine family
D10989	Bos taurus endothelin ETB receptor	2.12	1	
AB005148	Bos taurus interleukin 1 (IL-1) receptor antagonist	2.13	1	Cytokine family
AF192336	Bos taurus PAG-19	2.17	11	Cytokine family
BP110455	EST	2.18	1	
J02840	Bos taurus placental lactogen (PL-Ala)	2.21	5	Cytokine family
M73961	Ovis aries PAG-1	2.35	6	Cytokine family
AF004133	Sus scrofa adipocyte membrane protein	2.66	1	Membrane protein
AW463219	EST	2.77	5	
BP111699	EST	2.85	5	
X01912	Goat epsilon I beta-globin	3.00	9	
AF020508	Bos taurus PAG-6	3.45	11	Cytokine family

Supplement Table 4

Two-fold differentially expressed genes between Day 28EEM vs Day 28F.

Accession#	Gene name	D28F/D28M	k-means	classification
	Day 28F /Day 28EEM down-regulated genes (< 0.5)			
AW462349	EST	0.05	11	
AW484050	EST	0.12	11	
M73961	Ovis aries PAG-1	0.14	6	Cytokine family
X89984	Homo sapiens BCL7A protein	0.14	12	Cytoskelton
AA459491	EST	0.14	11	
X15975	Bos taurus PRP-V	0.15	11	Cytokine family
AW464858	EST	0.15	11	
BP111741	EST	0.15	11	
AF079545	Ovis aries placental lactogen precursor (PL)	0.16	8	Cytokine family
AF020509	Bos taurus PAG-7	0.19	11	Cytokine family
J02840	Bos taurus placental lactogen (bPL-Ala)	0.20	5	Cytokine family
X59504	Bos taurus PRP-VI	0.20	11	Cytokine family
S72871	Homo sapiens GATA-2 transcription factor	0.20	8	Transcriptional regulator
AW463993	EST	0.20	11	
M65219	Bos taurus placental lactogen (bPL-Ala)	0.22	5	Cytokine family
AF192336	Bos taurus PAG-19	0.22	11	Cytokine family
BP112760	EST	0.23	12	
AW464536	EST	0.23	5	
AF020508	Bos taurus PAG-6	0.23	11	Cytokine family
AA837482	EST	0.23	5	
BP107836	EST	0.24	11	
J02944	Bos taurus PRP-I	0.24	11	Cytokine family
BP112768	EST	0.24	12	
L06151	Bos taurus PAG-2	0.25	11	Cytokine family
AF020512	Bos taurus PAG-10	0.27	5	Cytokine family
AW465434	EST	0.27	11	
AW356791	EST	0.28	5	
AF020514	Bos taurus PAG-12	0.28	11	Cytokine family
AW464645	EST	0.28	3	
AW463668	EST	0.29	5	
BP112727	EST	0.29	11	
AW485575	EST	0.30	11	
AF192334	Bos taurus PAG-17	0.30	11	Cytokine family
AL133034	Homo sapiens clone DKFZp727K171	0.31	11	
Z11742	Bos taurus annexin XI	0.31	5	Apoptosis & Cell cycle
U89321	Homo sapiens nucleophosmin phosphoprotein (NPM)	0.32	5	
M25493	Bos taurus PRP-I	0.32	11	Cytokine family
M80328	Bos taurus bPL-Val	0.32	5	Cytokine family
X17614	Bos taurus 3 beta hydroxy-5-ene steroid dehydrogenase/delta 5-	0.32	5	
AF192333	Bos taurus PAG-16	0.32	11	Cytokine family
AF020507	Bos taurus PAG-5	0.34	11	Cytokine family
AW464661	EST	0.34	11	
AW465275	EST	0.34	6	
AW465912	EST	0.34	11	
AW462855	EST	0.34	11	
AF020511	Bos taurus PAG-9	0.35	5	Cytokine family
AF020510	Bos taurus PAG-8	0.35	3	Cytokine family
BP110551	EST	0.35	5	
AF106681	Homo sapiens ras-related GTP-binding protein	0.36	11	
BP110145	EST	0.37	8	
M33269	Bos taurus PRP-IV	0.37	3	Cytokine family
AF192332	Bos taurus PAG-15	0.37	11	Cytokine family
AW463219	EST	0.37	5	
NM_005720	Homo sapiens actin related protein 2/3 complex, subunit 1B	0.38	3	Cytoskelton
AW465464	EST	0.38	5	
NM_006429	Homo sapiens CCT7	0.39	11	
AW464023	EST	0.39	5	
BP111699	EST	0.39	5	
AW463677	EST	0.39	6	
K02130	Bos taurus cytochrome P-450 (SCC)	0.41	5	
AF004133	Sus scrofa adipocyte membrane protein	0.41	1	Membrane protein
D50047	Bos taurus KQ217 MHC class II DQA2	0.42	5	Cytokine family
NM_012198	Homo sapiens grancalcin (GCL)	0.42	3	
BP112100	EST	0.43	5	
AW461508	EST	0.43	5	
BP107921	EST	0.43	11	
AF080067	Oryctolagus cuniculus SMVT	0.43	6	
AW356197	EST	0.43	11	
L41543	Bos taurus epithelial mucin (MUC1)	0.43	11	Cell adhesion
AW462849	EST	0.44	11	
AF192330	Bos taurus PAG-13	0.46	5	Cytokine family
AF177394	Homo sapiens dickkopf-1 (DKK-1)	0.47	5	
BP107489	EST	0.47	5	
AW464053	EST	0.47	5	
	Day 28F / Day 28EEM up-regulated genes (2 <)			
AF161496	Homo sapiens HSPC147	2.00	9	
AW479517	EST	2.00	8	
AW345567	EST	2.01	12	
AI816701	EST	2.01	7	
AW426436	EST	2.01	12	
NM_004530	Homo sapiens MMP-2	2.01	7	ECM & related
L10325	Bos taurus glutathione peroxidase (GPx) plasma isoform	2.01	7	Enzyme
X53553	Bos taurus insulin-like growth factor II (IGF-II)	2.01	10	Cytokine family
AW425047	EST	2.01	8	
BP112833	EST	2.01	1	
D84557	Homo sapiens HsMcm6	2.02	12	
AW485951	EST	2.03	12	
BP111504	EST	2.04	8	
AW315092	EST	2.04	10	
NM_010064	Mus musculus Dncic2	2.04	3	Cytoskelton
U41668	Homo sapiens deoxyguanosine kinase	2.04	3	Enzyme
AW307840	EST	2.04	3	
AW314100	EST	2.04	10	
AF083246	Homo sapiens HSPC028	2.05	12	
AW432050	EST	2.05	9	
NM_001827	Homo sapiens CDC28 protein kinase 2 (CKS2)	2.05	8	Apoptosis & Cell cycle

X00354	Bos taurus gamma globin	2.05	7	
AF092565	Homo sapiens splicing factor Prp8	2.06	6	Transcriptional regulator
AF034607	Homo sapiens chloride channel ABP	2.07	8	Membrane protein
AF002693	Homo sapiens ATQL1 pseudogene	2.07	2	
AI326172	EST	2.07	12	
AJ000186	Homo sapiens MAD2 protein	2.07	7	Cytokine family
AI276594	EST	2.07	3	
AW427732	EST	2.08	3	
AB017335	Homo sapiens KNSL4 and MAZ	2.08	2	Apoptosis & Cell cycle
AI827165	EST	2.09	8	
AW481254	EST	2.09	10	
AF161447	Homo sapiens HSPC329	2.10	8	
AW345789	EST	2.10	9	
AF110801	Homo sapiens interleukin-18 binding protein c precursor	2.10	9	Cytokine family
AW315457	EST	2.11	9	
AF039698	Homo sapiens antigen NY-CO-33	2.11	7	Cytokine family
BP112517	EST	2.11	1	
AW427810	EST	2.11	10	
BP111255	EST	2.11	7	
U71273	Sus scrofa glucosidase II	2.12	6	Enzyme
BP110613	EST	2.12	8	
BP109873	EST	2.13	7	
BP109935	EST	2.15	7	
AC002550	Homo sapiens BAC clone CIT987SK-A-101F10	2.16	9	
AF086313	Homo sapiens clone ZD52B10	2.17	6	
AB004275	Bos taurus VEGF-C	2.17	1	Cytokine family
BP109933	EST	2.17	12	
AW353298	EST	2.18	6	
AW314471	EST	2.18	3	
BP109730	EST	2.18	2	
BP106960	EST	2.19	9	
AL137647	Homo sapiens clone DKFZp586M2424	2.19	10	
BP112666	EST	2.20	3	
D14074	Bos taurus cyclophilin	2.21	8	
BP112133	EST	2.21	1	
AW416500	EST	2.21	2	
U49973	Homo sapiens Tigger1 transposable element	2.22	9	
AW299830	EST	2.22	10	
NM_002130	Homo sapiens HMGCS1	2.23	6	Enzyme
AI952986	EST	2.23	8	
AW483954	EST	2.24	3	
AW344559	EST	2.24	4	
BP111358	EST	2.24	10	
U21661	Rattus norvegicus myotrophin	2.24	2	Cytokine family
AW427170	EST	2.24	7	
X13698	Bos taurus ribonucleoprotein SS-B/La	2.25	10	Nuclear protein
NM_001145	Homo sapiens RNase A family 5 (ANG)	2.26	10	Enzyme
BP109382	EST	2.26	7	
U19921	Homo sapiens synaptotagmin I	2.26	1	
X59693	Bos taurus ubiquinol-cytrochrome-c reductase (subunit II)	2.26	6	Enzyme
BP109023	EST	2.27	7	
NM_003011	Homo sapiens SET translocation	2.28	10	
AW462084	EST	2.31	8	
AJ132772	Bos taurus futb and rtlf	2.32	6	
BP107868	EST	2.33	2	
AF198487	Homo sapiens transcription factor LBP-1b	2.34	4	Transcriptional regulator
AW314471	EST	2.35	3	
NM_005563	Homo sapiens stathmin 1/oncoprotein 18 (STMN1)	2.36	4	Oncogene & Tumor inhibitor
AF144763	Bos taurus TIMP-1 protein	2.36	3	ECM & related
AF043937	Homo sapiens DHAPAT	2.37	9	Enzyme
M86739	Bos taurus neuropeptide Y receptor	2.38	7	Cytokine family
Y16533	Ovis aries IGF-II	2.39	7	Cytokine family
AW484739	EST	2.39	4	
S76474	Homo sapiens trkB	2.39	1	Cytokine family
AF113682	Homo sapiens clone FLB3436 PRO0868	2.40	4	
AW345891	EST	2.41	8	
Z25531	Bos taurus repeat region DNA	2.41	3	
BP108502	EST	2.43	9	
AW353230	EST	2.45	3	
AW477773	EST	2.49	10	
H27490	EST	2.49	10	
AF195417	Homo sapiens DEAD-box protein abstrakt (ABS)	2.51	2	
BP111076	EST	2.52	7	
AW446540	EST	2.52	9	
NM_006719	Homo sapiens transcript variant ABLIM-m	2.53	1	Cytoskelton
AW466068	EST	2.56	8	
AB043994	Bos taurus MMP-2	2.56	7	ECM & related
AW195100	EST	2.57	2	
BP107329	EST	2.62	4	
AF057300	Homo sapiens truncated RAD50 protein	2.63	3	
AW194656	EST	2.64	2	
AL080102	Homo sapiens clone DKFZp564N1916	2.64	9	
AB028449	Homo sapiens helicase-MOI	2.65	9	DNA binding protain
BP111624	EST	2.67	7	
NM_006838	Homo sapiens methionine methionyl aminopeptidase 2	2.72	4	Enzyme
AW411362	EST	2.75	9	
BP111800	EST	2.76	7	
AA453356	EST	2.79	3	
AT001971	EST	2.79	2	
NM_006491	Homo sapiens NOVA1 transcript variant 3	2.82	1	Oncogene & Tumor inhibitor
NM_004458	Homo sapiens FACL4 transcript variant 1	2.84	7	Enzyme
AF000137	Bos taurus connective issue growth factor precursor (CTGF)	2.85	12	Cytokine family
AW020030	EST	3.06	7	
AW485227	EST	3.15	10	
AW173315	EST	3.40	10	
AW486974	EST	3.58	10	
L34261	Bos taurus palmitoyl-protein thioesterase	4.32	6	Enzyme
BP108345	EST	4.60	7	
